# KLF12 promotes the proliferation of breast cancer cells by reducing the transcription of *p21* in a p53-dependent and p53-independent manner

**DOI:** 10.1038/s41419-023-05824-x

**Published:** 2023-05-08

**Authors:** Yanan Li, Shujing Li, Xiaoxia Shi, Zhiqiang Xin, Yuxi Yang, Binggong Zhao, Yvlin Li, Linlin Lv, Ping Ren, Huijian Wu

**Affiliations:** 1 School of Bioengineering & Key Laboratory of Protein Modification and Disease, Liaoning Province, Dalian University of Technology, 116024 Dalian, China; 2 The Second Hospital of Dalian Medical University, 116000 Dalian, China

**Keywords:** Biological sciences, Cancer

## Abstract

Breast cancer is the most common cancer affecting women worldwide. Many genes are involved in the development of breast cancer, including the *Kruppel Like Factor 12* (*KLF12*) gene, which has been implicated in the development and progression of several cancers. However, the comprehensive regulatory network of KLF12 in breast cancer has not yet been fully elucidated. This study examined the role of KLF12 in breast cancer and its associated molecular mechanisms. KLF12 was found to promote the proliferation of breast cancer and inhibit apoptosis in response to genotoxic stress. Subsequent mechanistic studies showed that KLF12 inhibits the activity of the p53/p21 axis, specifically by interacting with p53 and affecting its protein stability via influencing the acetylation and ubiquitination of lysine370/372/373 at the C-terminus of p53. Furthermore, KLF12 disrupted the interaction between p53 and p300, thereby reducing the acetylation of p53 and stability. Meanwhile, KLF12 also inhibited the transcription of *p21* independently of p53. These results suggest that KLF12 might have an important role in breast cancer and serve as a potential prognostic marker and therapeutic target.

## Introduction

Breast cancer is the leading cancer in women and the fourth cause of cancer-related death in China [1]. Surgery is the best treatment approach for primary breast cancer, while chemotherapy, endocrine therapy, and radiotherapy remain the most effective therapeutic methods for advanced-stage breast cancer. However, despite significant advances in early detection and steady progress in treating systemic agents, most patients are resistant to drugs. Over the years, understanding the underlying biological mechanisms of breast cancer has led to identifying novel molecular targets and developing targeted therapies.

The Kruppel-like family (KLF) of transcription factors involves multiple processes, such as proliferation, differentiation, migration, and pluripotency [2, 3]. KLF12 was first identified as a transcription factor for activating protein-2 (AP-2). KLF12 has a Pro-Xaa-Asp-Leu-Ser (PVDLS) motif at the N-terminus, allowing it to bind to the transcription corepressor C-terminal binding protein (CtBP). In addition, KLF12 binds to promotors of its target genes at the consensus sequence CAGTGGG [4, 5]. Increasing evidence has shown that KLF12 has an important role in several kinds of cancer by affecting different biological processes [6, 7]. Furthermore, KLF12 has been reported to be a tumor suppressor or inducer tightly dependent on the different signaling crosstalks or partners in a specific cellular environment [6–10]. However, the comprehensive regulatory network of KLF12 in cancer has not been fully elucidated.

More recently, the tumor suppressor microRNA-205 has been reported to directly target KLF12 and inhibit the invasion and apoptosis of basal-like breast carcinoma (BLBC), suggesting that KLF12 may be a potential biomarker of BLBC [11, 12]. In addition, through Expression Data Analysis, Rajeswary and coworkers have verified KLF12 as a potential target for controlling breast cancer development without harming pregnancy and the fetal developmental process [13]. These findings suggest that KLF12 may have an important role in breast cancer. However, these studies lack direct experimental data. Thus, exploring the molecular mechanism of KLF12 in breast cancer may provide new information for improving breast cancer treatment.

The tumor suppressor p53 functions as a transcription factor and regulates downstream targets responsible for homeostasis and defense against tumorigenesis [14]. KLF4, a member of the KLF family, can interact with p53 and mediate the transcription of *p21* [15]. KLF9, another member of the KLF family, can inhibit the growth of hepatocellular carcinoma cells and cause apoptosis by inducing the transcription of *p53* [16]. Besides, genome-wide analysis of p53 ChIP-Seq has predicted KLF12 to be a co-regulator with p53 [17]. Therefore, we speculated that KLF12 might regulate the occurrence and development of breast cancer through the p53 signaling pathway. As a cellular gatekeeper for growth, p53 mediates the events in the cell cycle and apoptosis by regulating the expression of downstream target genes [18–20], such as *p21*, *GADD45*, *PUMA*, and *cyclin G* [21, 22]. p21 is an important inhibitor of cyclin-dependent kinases (CDKs) and in the regulation of CDK activity and cell cycle progression, p21 has also been found to be involved in cell differentiation, senescence, DNA repair, and apoptosis [23, 24]. In breast cancer, p21 is regarded as a protein with dual functions, as it is thought to be the chief mediator of p53-dependent cell cycle arrest caused by a stimulus, such as DNA damage, oncogene activation, and hypoxia [24]. Cisplatin (CDDP), a chemotherapeutic agent commonly used in the clinic, can cause DNA damage and induce the tumor cells to undergo apoptosis with increased expression of p53 and p21 [25, 26]. However, p21 may also be an oncogene because it can inhibit cell apoptosis [27]. Therefore, careful planning is needed if p21 is the intended target of the therapy for breast cancer patients.

Post-translational modifications (PTMs) of p53, such as methylation, acetylation, SUMOylation, and ubiquitination, have important roles in regulating the transcriptional activity and protein stability of p53 [28]. p53 is subject to acetylation by p300/CBP at multiple lysine residues (residues 370, 372,373, 381, 382, and 386) [29]. Lys^216^ and Lys^305^ were later found to be acetylated by p300/CBP [30, 31]. These six lysine residues (Lys 370, 372,373, 381, 382, and 386) can also be ubiquitinated. Acetylation at these sites prevents p53 from being ubiquitinated, thus increasing its stability [31, 32].

In this study, we examined the role of KLF12 in breast cancer and its associated molecular mechanisms. The results showed that KLF12 could promote the proliferation of breast cancer cells and inhibit the apoptosis of cancer cells treated with CDDP. Such effects of KLF12 on breast cancer cells appeared to stem from its ability to inhibit the stability of p53 by reducing p300-mediated acetylation of p53 and to directly inhibit the transcription *p21* by binding to the promoter of *p21*. This data may further our understanding of the molecular mechanism of KLF12 in breast cancer.

## Results

### KLF12 has an important role in the proliferation, cell cycle, and apoptosis of breast cancer cells

**Fig. 1:**
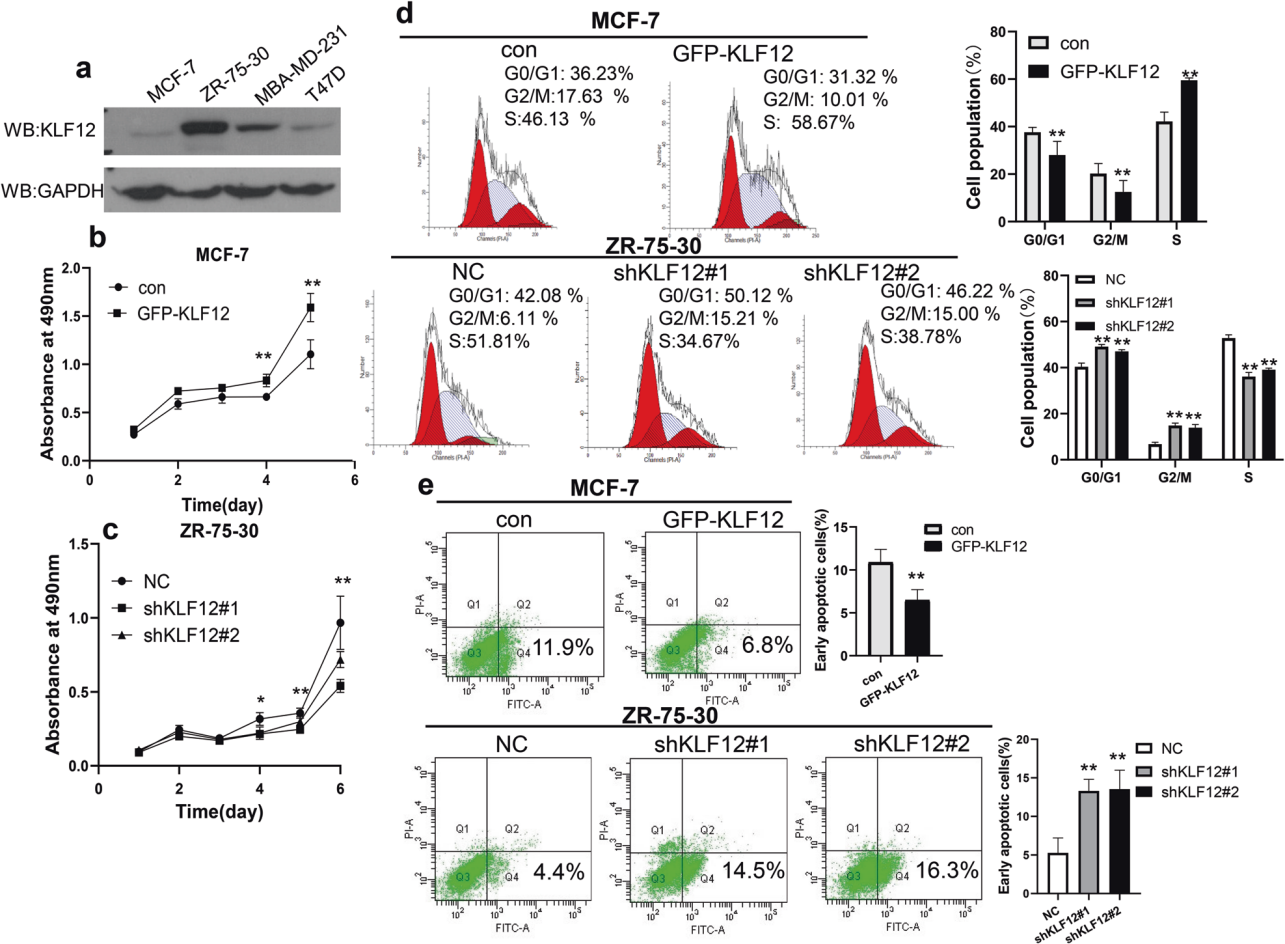
KLF12 has an important role in breast cancer’s proliferation, cell cycle, and apoptosis. **a** The expression of KLF12 in Hela, MCF10A, ZR-75-30, MCF-7, MBA-MD-231, and T47D cells by western blot assay. **b** MTT assay showing the growth curves of MCF-7 cells transfected with GFP-KLF12 or the control vector. Data are presented as means ± SDs. (*p* < 0.05, significant; ns, not significant; *p < 0.05; **p < 0.01). **c** MTT assay showing the growth curves of ZR-75-30 cells transfected with NC, shKLF12#1, and shKLF12#2. Data are presented as means ± SDs. (*p* < 0.05, significant; ns, not significant; *p < 0.05; **p < 0.01). **d** The effect of KLF12 on the cell cycle of breast cancer cell lines by flow cytometry assay. MCF-7 cells were transfected with GFP-KLF12 or the control vector. ZR-75-30 cells were transfected with NC, shKLF12#1, and shKLF12#2. The histogram on the right shows the percentage of different phase cells. **e** The effect of KLF12 on cellular apoptosis by flow cytometry assay. MCF-7 cells were transfected with GFP-KLF12 or the control vector. ZR-75-30 cells were transfected with NC, shKLF12#1, and shKLF12#2. The histogram on the right shows the percentage of early apoptotic cells.

Previous studies have shown that KLF12 may have an important role in breast cancer [11, 13]. To further investigate the role of KLF12 in breast cancer, we first examined the protein levels of KLF12 in breast cancer cells (MCF-7, ZR-75-30, T47D, and MDA-MB-231). A higher level of KLF12 was found in ZR-75-30, MDA-MB-231 cells, and T47D cells compared with MCF-7 cells (Fig. 1a).

Next, the effect of KLF12 on the growth and tumorigenesis of breast cancers was determined by evaluating its effect on the viability of the cells using an MTT assay. A significant increase in cell viability was detected in MCF-7 cells that overexpressed KLF12 compared with those that did not overexpress KLF12 (Fig. 1b). After knocking down the expression of KLF12, the viability of ZR-75-30 cells was reduced (Figs. 1c and S1a).

To further consolidate this observation, colony formation was performed. The results showed that overexpression of KLF12 resulted in a marked increase in cell number, while the knockdown of KLF12 decreased the colony formation (Fig. S1b). Collectively, the results suggested that KLF12 could promote the growth of breast cancer cells.

These cells were further subjected to cell cycle and apoptosis assays. Flow cytometry analysis demonstrated that overexpression of KLF12 in MCF-7 cells reduced the number of cells in the G_0_/G_1_ phase and G2/M phase and increased the number of cells in the S phase (Fig. 1d). Conversely, the knockdown of KLF12 expression in ZR-75-30 cells increased the number of cells in the G_0_/G_1_ phase and G2/M (Fig. 1d). Notably, the percentage of early apoptotic cells significantly decreased in KLF12-overexpressing MCF-7 cells when treated with CDDP (Fig. 1e). Meanwhile, the percentage of early apoptotic cells was significantly increased in ZR-75-30 cells with KLF12 knockdown (Fig. 1e). Collectively, these results suggested that KLF12 might have an important role in promoting growth and inhibiting apoptosis in breast cancer cells.

### KLF12 is a transcription repressor of *p21* that inhibits p53-mediated transcriptional activation of *p21*

**Fig. 2:**
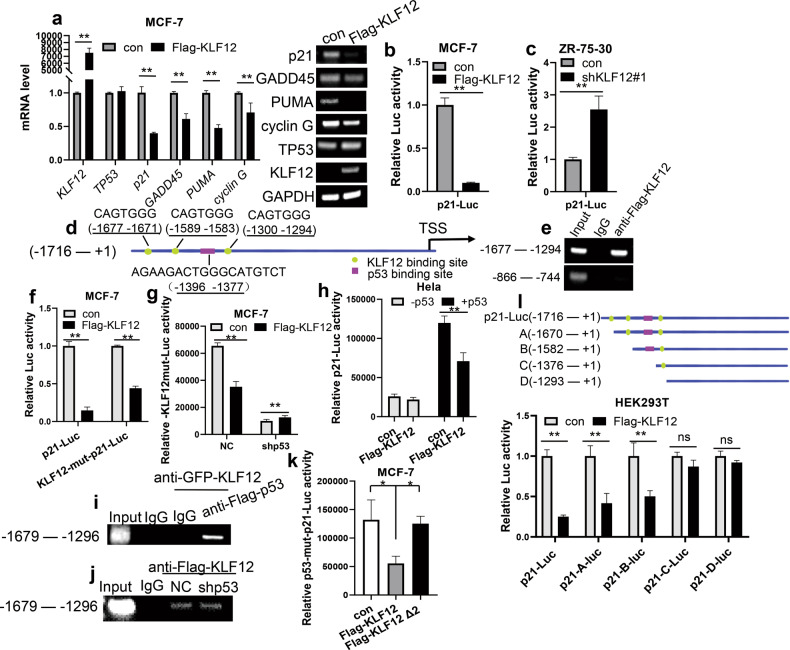
KLF12 is a transcription repressor of p21 and inhibits p53-mediated transcriptional activation of p21. **a** mRNA levels of p53 target genes *p21*, *GADD45*, *PUMA*, and *cyclin G* in MCF-7 cells with overexpression of Flag-KLF12 or vector detected by RT-PCR assay. Data are presented as means ± SDs. (*p* < 0.05, significant; ns, not significant; *p < 0.05; **p < 0.01). **b**, **c** Luciferase report assay tested the effect of KLF12 on the reporter activity of p21-Luc in MCF-7 cells and ZR-75-30 cells. For comparison, the p21-Luc activity level of control cells was set to 1. Data are presented as means ± SDs (*p* < 0.05, significant; ns, not significant; *p < 0.05; **p < 0.01). **d** Schematic diagram of the p21 promoter-Luc. **e** ChIP assays demonstrated the binding of KLF12 to the *p21* promoter. An anti-Flag antibody was used to pull down Flag-KLF12 in HEK293T cells. IgG was used as a negative control. Nucleotides −1677 to −1294 of the *p21* promoter contain the conserved binding site for KLF12. The sequence containing nucleotides −866 to – 744 was used as a negative control in the PCR amplification. **f** Luciferase report assay tested the effect of KLF12 on KLF12-mut-p21-Luc. Luciferase activity was measured after 24 h of transfection. For comparison, the activity levels of p21-Luc and p21-KLF12-mut-Luc of the control cells were set to 1. Data are presented as means ± SDs (*p* < 0.05, significant; ns, not significant; *p < 0.05; **p < 0.01). **g** Luciferase activity assay tested the effect of KLF12 on KLF12-mut-p21-Luc in the presence of p53 knockdown. Data are presented as means ± SDs (*p* < 0.05, significant; ns, not significant; *p < 0.05; **p < 0.01). **h** Luciferase activity assay tested the effect of KLF12 on p53 transcription activity in Hela without or with overexpression of exogenous p53. Data are presented as means ± SDs (*p* < 0.05, significant; ns, not significant; *p < 0.05; **p < 0.01). **i** ChIP-Re-ChIP assay showed the co-localization of KLF12 with p53 on *p21* promoter (−1679 to −1296) in HEK293T cells. DNA protein complexes were subjected to two sequential immunoprecipitation assays, first with an anti-GFP antibody to pull down GFP-KLF12 and then with an anti-Flag antibody to pull down Flag-p53 the second round of ChIP. **j** ChIP assay showed the effect of p53 knockdown on the binding of KLF12 to the *p21* promoter. An anti-Flag antibody was used to pull down Flag-KLF12 in HEK293T cells. **k** Luciferase activity assay tested the effect of KLF12 on the reporter activity of p53-mut-p21-Luc in MCF-7 cells. For comparison, the activity level of p53-mut-p21-Luc in the control cells was set to 1. Data are presented as means ± SDs (*p* < 0.05, significant; ns, not significant; *p < 0.05; **p < 0.01). **l** Luciferase activity assay tested the effect of KLF12 on the different versions of truncated p21-Luc mutants in MCF-7 cells. For comparison, the activity level of the truncated p21-Luc mutant in the control cells was set to 1. Data are presented as means ± SDs (*p* < 0.05, significant; ns, not significant; *p < 0.05; **p < 0.01).

The molecular mechanisms underlying the proliferative and anti-apoptotic effect of KLF12 were further explored. Previous studies have predicted that KLF12 may be a co-regulator of p53 [17]. Therefore, we hypothesized that KLF12 might regulate the development of breast cancer cells through the p53 signaling pathway. To verify our hypothesis, qPCR was used to check the mRNA levels of *TP53* and p53 downstream target genes in KLF12-overexpressing MCF-7 cells and KLF12-knockdown ZR-75-30 cells. The mRNA levels of p53 target genes, such as *p21*, *GADD45*, *PUMA*, and *cyclin G* decreased while the mRNA level of *TP53* remained relatively unchanged in KLF12-overexpressing MCF-7 cells (Figs. 2a and S2a), demonstrating that KLF12 down-regulate the transcription of p53 target genes. Consistent with this result, the mRNA levels of p53 target genes increased while the mRNA level of *TP53* remained unchanged in KLF12-knockdown ZR-75-30 cells (Fig. S2b, c).

As a CDK inhibitor and a classical gene transcriptionally regulated by p53, p21 bridges the function of a tumor suppressor with the cell cycle [33]. Following the activation of p53, e.g., by a viral infection or induction of DNA damage, the *p21* gene is transcriptionally activated by p53 [34]. Therefore, we next assessed the effect of KLF12 on the p53/p21 signal axis via luciferase reporter assay. The activity of p21-Luc was inhibited in MCF-7 cells that overexpressed KLF12 (Fig. 2b), while the activity of p53-Luc did not significantly change (Fig. S2d). Contrary, the knockdown of KLF12 expression in ZR-75-30 cells resulted in increased activity of p21-Luc in these cells (Fig. 2c). Taken together, KLF12 could down-regulate the mRNA level of *p21* by transcriptionally suppressing the activity of its promoter.

To investigate the mechanism through which KLF12 down-regulates the activity of the *p21* promoter, we checked for a KLF12-binding site on the *p21* promoter. Three KLF12 binding sites (−1677 to −1671, −1589 to −1583, − 1300 to −1294) close to the binding site of p53 (−1396 to −1377) were found on the *p21* promoter (Fig. 2d). ChIP experiment was then conducted, and the result showed that KLF12 could bind to the *p21* promoter (Fig. 2e). However, when all three KLF12-binding sites were mutant, a dramatic drop in the inhibitory effect of KLF12 occurred, yielding an approximately 30% decrease in the inhibition of *p21* transcription. Nonetheless, the inhibition did not completely disappear (Fig. 2f), which means the KLF12-binding sites may not be the only way KLF12 inhibits the transcription of *p21*. Previous studies have shown that KLF12 may be the co-regulator of p53. Therefore, we suspected that KLF12 could inhibit the transcription of *p21* through p53. Knocking down the expression of p53 abolished the KLF12-mediated suppression of KLF12-mut-p21-Luc activity (Figs. 2g and S2e). Moreover, KLF12 also inhibited p53-mediated transcriptional activation of *p21* (Fig. 2h). These findings confirmed that KLF12 inhibits the transcription of *p21* by affecting p53.

To further verify that KLF12 is a co-regulator of p53, a ChIP-Re-ChIP assay was carried out using HEK293T cells. The result indicated that KLF12 and p53 were co-located with the *p21* promoter, indicating that KLF12 may interact with p53 on the promoter of *p21* (Fig. 2i). Yet, the knockdown of p53 did not affect the binding of KLF12 to the *p21* promoter, suggesting that the binding of KLF12 to the *p21* promoter was independent of p53 (Fig. 2j). Subsequent reporter gene assays further indicated that KLF12 could inhibit the activity of p53-mut-p21-Luc with p53 binding sequence mutation, which means KLF12 could inhibit *p21* transcription independently of p53 (Fig. 2k). Besides, deletion of the ZNF (Zinc-finger) domain in KLF12 (Flag-Δ2KLF12) abolished the KLF12-mediated inhibition of p53-mut-p21-Luc (Fig. 2k). To sum up, KLF12 inhibits *p21* transcription in both p53-dependent and p53-independent ways.

To further explore this hypothesis, a series of p21-Luc truncated constructs (Fig. 2l) were used to verify the specific binding sites through which KLF12 could regulate the transcription of *p21*. The p21-Luc lost about 75% of the reporter activity in the presence of KLF12 overexpression. Deletion of KLF12-binding site 1 (−1677 to −1671) led to an approximately 15% decrease in KLF12 inhibitory activity; deletion of KLF12-binding site 1 (−1677 to −1671) and KLF12-binding site 2 (−1589 to −1583) led to a 27% decrease in KLF12 inhibitory activity. Interestingly, the deletion of KLF12-binding sites 1 and 2 and the p53-binding site (−1396 to −1377) resulted in an almost complete loss of KLF12 inhibitory activity. The above experiments indicate that KLF12 can inhibit p21-Luc activity through KLF12-binding sites 1 and 2 as well as the p53-binding site on the *p21* promoter (Fig. 2l). Thus, KLF12 could act as a co-regulator of p53 and regulate the transcription of *p21* through its effect on p53.

### KLF12 interacts with p53

**Fig. 3:**
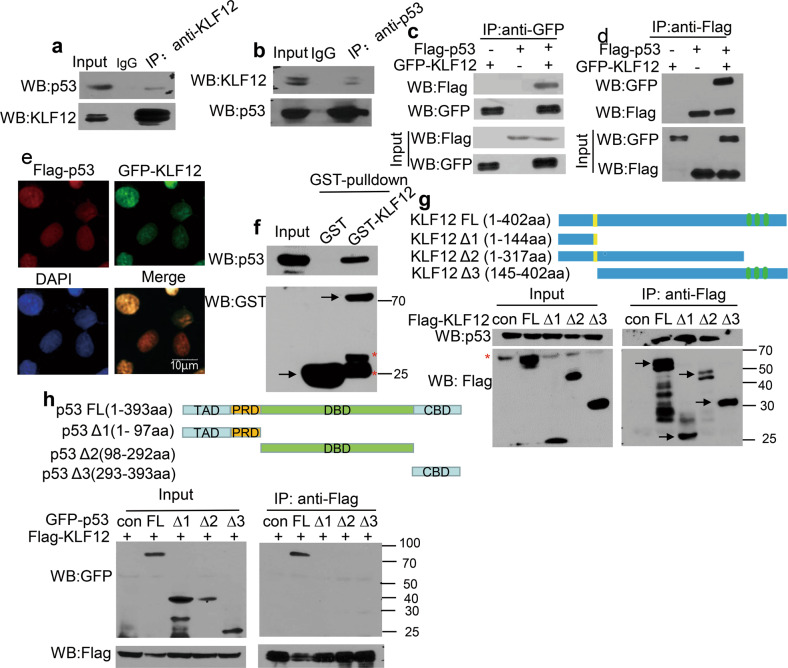
KLF12 interacts with p53. **a**, **b** CoIP assay detected an interaction between endogenous KLF12 and p53 in ZR-75-30 cells. **c**, **d** CoIP assay detected interaction between exogenous KLF12 and p53 in HEK293T cells. **e** Immunofluorescence assay showed the co-localization of GFP-KLF12 (green) with Flag-p53 (red) in the nucleus (blue) of Hela. **f** GST pull-down assay detected physical interaction between KLF12 and endogenous p53 in MCF7 cells. **g** CoIP assay identified the region of KLF12 that is required for interaction with p53. **h** CoIP assay identified the region of p53 that is required for interaction with KLF12.

The ability of KLF12 to inhibit the transcription of *p21* via p53 and co-localization with p53 on the *p21* promoter led us to hypothesize that KLF12 might inhibit the transcriptional activity of p53 by interacting with p53. To confirm this hypothesis, we performed co-immunoprecipitation (CoIP) assay in ZR-75-30 (Fig. 3a, b) and HEK293T cells (Fig. 3c, d). The results showed an interaction between endogenous and exogenous KLF12 and p53. Immunofluorescence and GST-pulldown assays were conducted to further verify the interaction between KLF12 and p53. Immunofluorescence assay demonstrated that KLF12 co-localized with p53 in the nucleus (Fig. 3e), and GST-pulldown revealed evidence of direct interaction between purified GST-KLF12 and endogenous p53 in MCF-7 cells (Fig. 3f). Moreover, to validate which region of the two proteins was involved in the interaction, mutants of KLF12 and p53 were constructed (Fig. 3g, h). The CoIP results indicated that KLF12 interacted with p53 through its N-terminal 1–144aa (Fig. 3g), and only full-length p53 could interact with KLF12 (Fig. 3h). Taken together, these results indicate the existence of physical interaction between KLF12 and p53 in breast cancer cells.

### KLF12 reduces the stability of p53

**Fig. 4:**
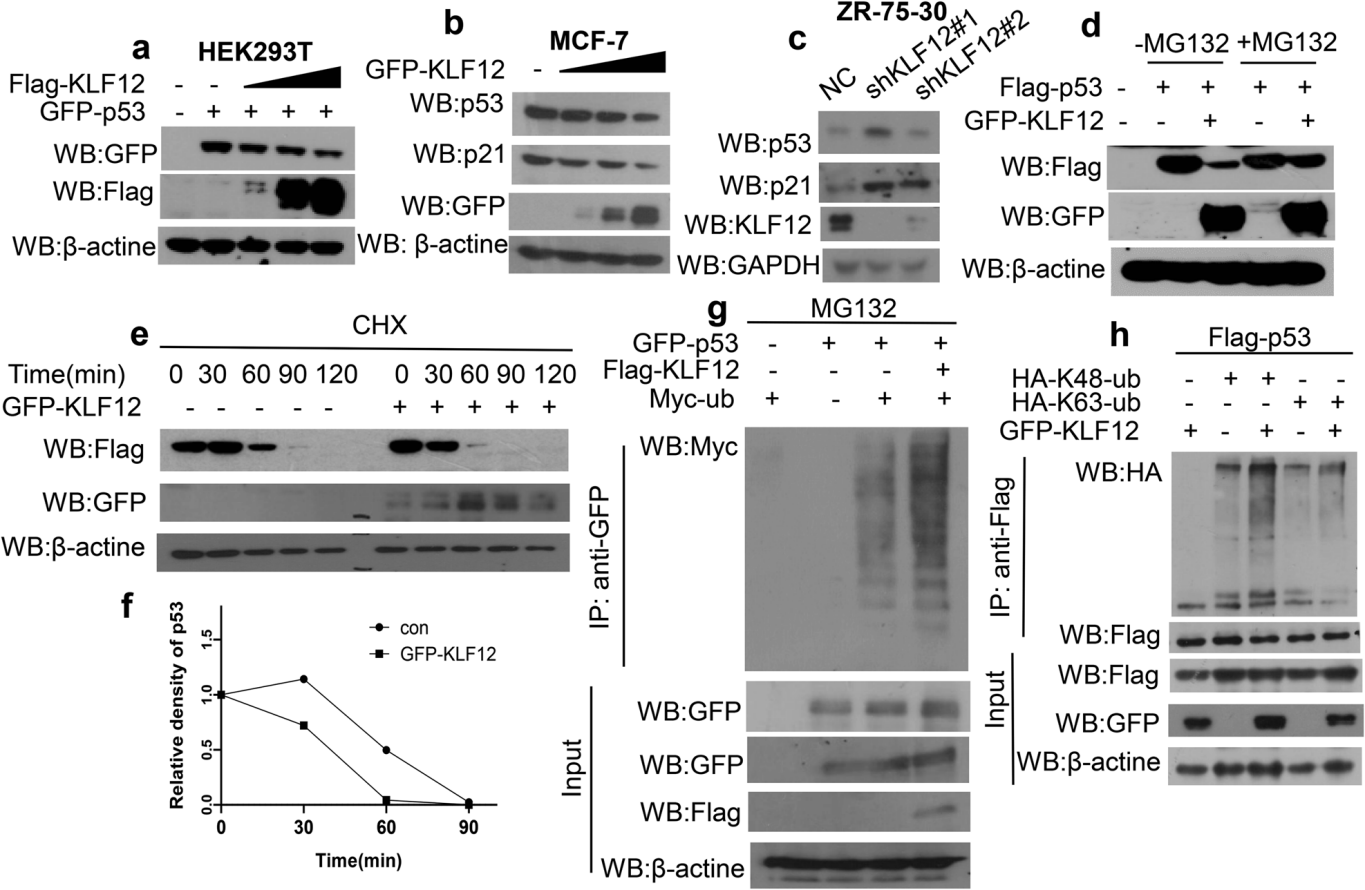
KLF12 reduces the stability of p53. **a** Western blot assay showed changes of exogenous p53 in HEK293T cells transfected with increasing doses of KLF12. **b** Changes in endogenous p53 and p21 in MCF7 cells transfected with increasing doses of KLF12 by Western blot assay. **c** Changes in the endogenous level of p53 and p21 in ZR-75-30 cells transfected with NC, shKLF12#1, or shKLF12#2 by Western blot assay. **d** Changes in exogenous p53 in HEK293T cells transfected with GFP-KLF12 without and with 20 μg/ml MG132 treatment by Western blot assay. **e**, **f** The half-life of p53 in MCF-7 cells with overexpressed KLF12 treated with CHX (50 μg/ml) by Western blot assay. **g** CoIP assay showed changes in the level of ubiquitinated p53 in HEK293T cells transfected with Flag-KLF12 plus 4 h treatment with 20 μg/ml MG132. **h** CoIP assay detected changes in K48-linked polyubiquitylation of p53 and K63-linked polyubiquitylation of p53 in HEK293T cells. The cells were treated with MG132 for 4 h following transfection.

To further establish the detailed mechanism through which KLF12 could inhibit p53-mediated transcription of *p21*, the influence of KLF12 on the protein level of p53 was first determined. A dose-dependent reduction of exogenous p53 occurred with increasing doses of KLF12 in HEK293T cells (Fig. 4a). Similarly, endogenous p53 and p21 also decreased in MCF-7 cells with overexpressed KLF12 (Fig. 4b). On the other hand, knocking down the expression of KLF12 in ZR-75-30 cells increased protein levels of endogenous p53 and p21 (Fig. 4c). Subsequent experiment showed that the reduction of p53 in the cells with overexpressed KLF12 could be prevented by treating cells with the proteasome inhibitor MG132 (Fig. 4d), which suggested that KLF12 could reduce the stability of p53. Meanwhile, the half-life of p53 was shorter in MCF7 cells with overexpressed KLF12 (Fig. 4e, f). These results indicate that KLF12 can probably affect the stability of p53 through the ubiquitin-proteasome pathway. To further verify this speculation, the effect of KLF12 on the ubiquitination system-mediated degradation of p53 was investigated.

Furthermore, a significant rise in ubiquitinated p53 was observed in HEK293T cells with overexpressed KLF12 (Fig. 4g). K48-Ub and K63-Ub ubiquitin linkages are the two main forms of ubiquitin chains [35]. Overexpression of KLF12 mainly induced the accumulation of K48-linked poly-ubiquitylated p53, while no visible change was detected for the level of K63-linked poly-ubiquitylated p53 (Fig. 4h). Taken together, these results suggested that KLF12 could effectively reduce its stability and promote the degradation of p53.

### KLF12 inhibits p300-catalyzed acetylation of p53

**Fig. 5:**
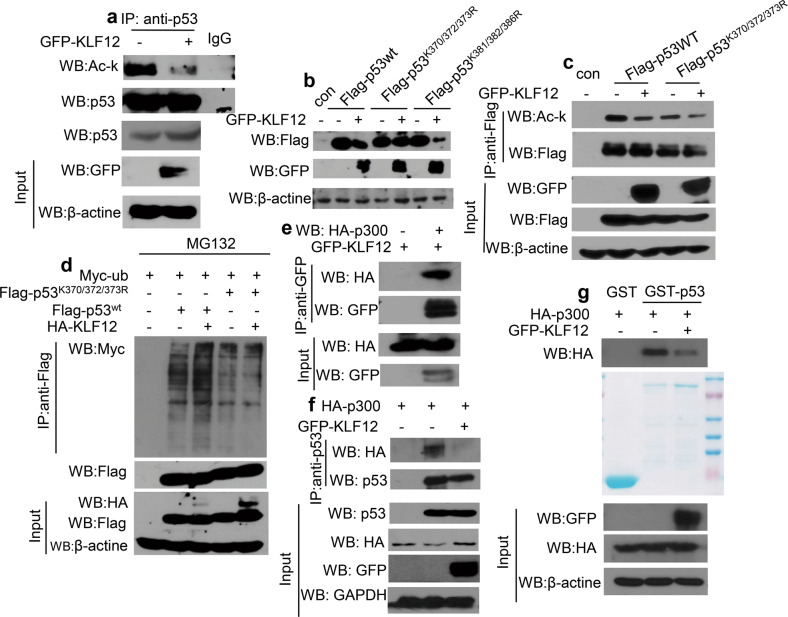
KLF12 reduces p53 protein levels by inhibiting the acetylation of p53 by p300. **a** CoIP assay showed the effect of KLF12 on the acetylation of endogenous p53 in MCF-7 cells. The endogenous p53 was coimmunoprecipitated with anti-p53 and then subjected to western blot analysis. **b** The effect of KLF12 on the protein levels of wild-type p53, p53^K370/372/373R,^ and p53^K381/382/386R^ in HEK293T cells by Western blot assay. **c** The effect of KLF12 on the acetylation of p53 and p53^K370/372/373R^ in HEK293T cells by CoIP assay. Flag-p53 and Flag-p53^K370/372/373R^ were coimmunoprecipitated with anti-Flag antibodies and then subjected to western blot analysis. **d** CoIP assay showed the changes in ubiquitin levels linked to Flag-p53 and Flag-p53^K370/372/373R^ in HEK293T cells. **e** CoIP assay showed an interaction between exogenous KLF12 and p300 in HEK293T cells. GFP-KLF12 was coimmunoprecipitated with anti-GFP and then subjected to western blot analysis. **f** CoIP assay detected the effect of KLF12 on the interaction between p300 with p53 in HEK293T cells. HEK293T cells were transfected with indicated plasmids. **g** GST-pulldown assay showed KLF12 inhibited the interaction of purified GST-p53 with p300 in vitro. HEK293T cells were transfected with indicated plasmids.

The mechanism associated with KLF12-promoted ubiquitination and degradation of p53 was further explored. Ubiquitination of lys 370, 372, 373, 381, 382, and 386 at the C-terminus of p53 mediate the degradation of p53. Also, acetylation at these sites will prevent p53 from being ubiquitinated, thus increasing its stability [31]. Considering that KLF12 might act as a co-regulator of p53, we hypothesized that KLF12 could regulate the protein stability of p53 by blocking the acetylation of p53. To test this hypothesis, the acetylation level of endogenous p53 in MCF-7 cells was determined by CoIP assay. Overexpression of KLF12 reduced the acetylation level of p53 (Fig. 5a).

Next, we verified whether KLF12 could reduce the acetylation of the lysine site at the C terminal of p53. Two p53 mutants (Flag-p53^K370/372/373R^ and Flag-p53^K381/382/386R^) were constructed. Western blot analysis showed that the protein level of Flag-p53^K370/372/373R^ was not affected by KLF12, but the protein level of Flag-p53^K381/382/386R^ and the protein level of wild-type p53 were reduced by KLF12 (Fig. 5b). Thus, KLF12 reduced the stability of p53 depends on lysine 370, K372 and K373 of p53. Replacing K370, K372, and K373 with arginine abolished the reduction of acetylation and ubiquitination of p53 (Fig. 5c, d). This suggests that KLF12 affects the protein stability of p53 by regulating its acetylation and ubiquitination of lysine 370/372/373.

p300/CBP are known to regulate the acetylation of p53, which are recruited to the *p21* promoter by p53 to co-regulate the transcription of *p21* [36]. The acetylation of p53 catalyzed by p300 can enhance the DNA-binding activity of p53 and stabilize it by preventing its ubiquitination at C-terminal [37, 38]. Thus, we hypothesized that KLF12 could probably block the p300-catalyzed acetylation of p53 by interfering with the interaction between p53 and p300. Such an action would require the interaction between KLF12 and p300. CoIP assay demonstrated the interaction between KLF12 and p300 (Fig. 5e). The interaction between p53 and p300 was also analyzed in HEK293T cells. CoIP assay revealed a reduced interaction between p53 and p300 in HEK293T cells with overexpressed KLF12, indicating that KLF12 interfered with the interaction between p53 and p300 (Fig. 5f). Besides, GST-pull down assay revealed KLF12 also inhibited p53 interaction with p300 in vitro (Fig. 5g). In conclusion, the above results indicated that KLF12 could negatively regulate the transcriptional activity and stability of p53 by reducing acetylation of p53 catalyzed by p300.

### KLF12 promotes the proliferation of breast cancer cells in vivo

**Fig. 6:**
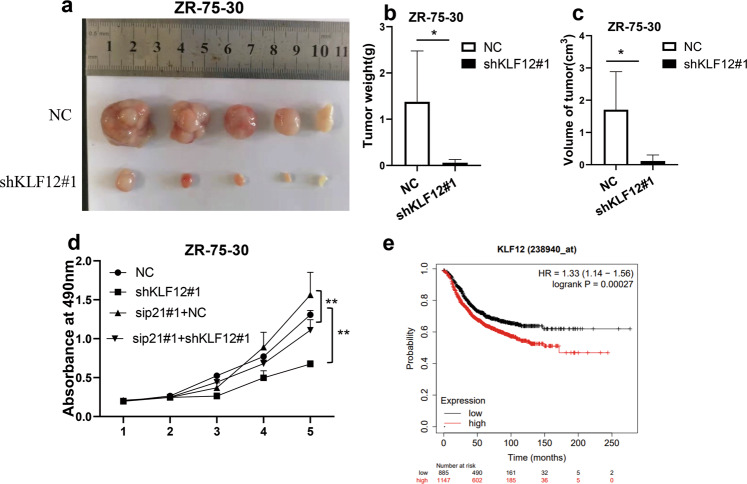
KLF12 promotes the proliferation of breast cancer cells in vivo. Five-week-old female BALB/C nude mice were inoculated with ZR-75-30 cells transfected with indicated plasmids; cells were injected in the right mammary fat pad. After 45 days, the mice were sacrificed, and the tumors were dissected, photographed, and weighed. **a** Image showing the sizes of tumors in xenograft mice. **b**, **c** Weight and volume of tumor-induced in mice by subcutaneously implanted ZR-75-30 cells, in which the KLF12 had been knockdown by shKLF12#1 or NC. Data are presented as means ± SDs (*p* < 0.05, significant; ns, not significant; *p < 0.05; **p < 0.01). **d** MTT assay showed the growth curves of ZR-75-30 cells with the indicated plasmid. Data are presented as means ± SD. (*p* < 0.05, significant; ns, not significant; *p < 0.05; **p < 0.01). **e** Kaplan–Meier curve of relapse-free survival times of breast cancer patients stratified by KLF12 expression levels. *p* = 0.00027 < 0.05. These data were obtained from http://kmplot.com/analysis/.Statistical significance was determined by the log-rank test.

The results obtained from cell culture experiments suggest that KLF12 may specifically repress the p53/p21 axis and promote the proliferation of breast cancer cells. Human breast cancer xenograft mouse model was constructed to further investigate the role of KLF12 in tumorigenesis. Mice implanted with ZR-75-30 cells in which the KLF12 expression was knockdown by shKLF12#1 exhibited smaller tumor volume and weight than the control mice (Fig. 6a–c). Besides, the MTT assay revealed that the cell proliferation-inhibiting role of the knockdown of KLF12 could be attenuated following the knockdown of p21 (Figs. 6d and S2e). The above data suggest that KLF12 could partially play a role in cell proliferation through p21.

To further investigate the effect of KLF12 on breast cancer patients, the relationship between KLF12 expression levels and the prognosis of breast cancer patients was then analyzed using the Kaplan-Meier Plotter database. The results showed that patients (1147 individuals) with high KLF12 expression experienced a shorter relapse-free survival time (RFS) and poorer prognosis than patients (885 individuals) with low KLF12 expression (Fig. 6e). Taken together, these results indicate an oncogenic effect of KLF12 with respect to the proliferation and tumorigenesis of breast cancer cells with wild-type p53, both at the cell and whole organism level. A high level KLF12 expression in the cancer cells or tissue may indicate a poor prognosis.

## Discussion

Many studies have suggested that KLF12 may contribute to tumor development in many types of carcinoma, and that its expression level is closely associated with the progression of malignancy and prognosis [6, 8, 39–41]. Previous studies have also shown that circ-NOTCH3 and the long non-coding RNA NEAT1 can promote breast cancer development by up-regulating KLF12 expression [12]. However, in this study, bioinformatics analysis using public datasets GEPIA (http://gepia.cancer-pku.cn) revealed no significant differences in KLF12 expression between breast cancer and normal breast tissues (Fig. S2g). To further investigate the role of KLF12 in the proliferation of breast cancer cells, we carried out extensive experiments. Our data revealed a positive effect of KLF12 on breast cancer proliferation, cell cycle, and apoptosis (Fig. 1a–e).

A dual function of p21Cip1/Waf1 was observed in carcinogenesis. In the presence of low doses of DNA damage, p21 causes cell cycle arrest and inhibits apoptosis, allowing DNA repair. After extensive DNA damage, the amount of p21Cip1/Waf1 protein is decreased, and the cell undergoes apoptosis [42]. While, there still some other studies have shown that up-regulated p21 expression promotes apoptosis [43, 44]. Hence, it is possible that KLF12 inhibit apoptosis by regulating p21 expression, but this needs further experimental verification. Besides, better understanding of the role of p21Cip1/Waf1 in various conditions would help to develop better cancer-treatment strategies. Moreover, studies have also shown that besides regulating p21, KLF12 also inhibit the expression of PUMA, an anti-apoptotic factor (Fig. 2a). In the presence of extensive DNA damage, cells initiate apoptosis through pro-apoptotic proteins downstream of p53, including PUMA. Therefore, KLF12 may inhibit apoptosis through PUMA, which also needs further experimental verification.

Furthermore, previous studies have predicted that KLF12 may be a co-regulator of p53 [17]. Consistently, we found that the overexpression of KLF12 could inhibit the expression of p53-targeted genes related to cell cycle and apoptosis (Fig. 2a), indicating that KLF12 could modulate the p53 pathway. Unlike KLF9 [16], KLF12 significantly inhibits the transcription of *p21* but does not affect the level of *p53* mRNA (Figs. 2a and S2d). Subsequent experiments identified KLF12 as a transcription factor of *p21*. KLF12 could inhibit the transcription of *p21* through its transcriptional activity (Fig. 2k). At the same time, KLF12 inhibited the p53-mediated transcriptional activation of *p21* (Fig. 2f–h). In addition, direct interaction between KLF12 and p53 might even occur on the *p21* promoter since the Ch-Re-ChIP assay showed the co-localization of KLF12 and p53 on the *p21* promoter (Fig. 2i). Such interaction between KLF12 and p53 was also demonstrated by the subsequent CoIP assay (Fig. 3a–c). Further investigation revealed a reduced level of p53 as a result of enhanced p53 ubiquitination facilitated by the overexpression of KLF12 (Fig. 4g, h). Since p53 is a tumor suppressor that regulates downstream targets responsible for homeostasis and defense against tumorigenesis [45], reducing the level of p53 could be a way for KLF12 to promote tumor proliferation and resist apoptosis.

Previous studies have shown that Yin Yang 1 (YY1) can inhibit p300-mediated acetylation and stabilization of p53 [46], while human ssDNA-binding protein SSB1 (hSSB1) can interact with p53 and protect p53 from ubiquitin-mediated degradation by recruiting p300 to p53 [47]. These two studies suggest that the stability of p53 is regulated by proteins that can affect its modification and alter its susceptibility to protease-mediated degradation. The acetylated lysine residues in p53 are located in its C-terminus and coincide with residues important for the protein’s ubiquitination and degradation [27, 45]. Similar to YY1, KLF12 could also inhibit the acetylation of p53 by interacting with p300, thereby blocking the stabilization and accumulation of p53 (Fig. 5a, b, g). Thus, p300 could be the critical protein through which KLF12 might regulate the transcriptional activity, acetylation, ubiquitination, and stability of p53. Unlike hSSB1, KLF12 was found to affect the transcriptional activity and protein stability of p53 by inhibiting the acetylation at lys370, lys372, and lys373 rather than the acetylation at lys382 (Fig. 5b–d).

Acetylation of p53 is a dynamic process. In addition to acetylase, deacetylases, including HDACs and SIRTs, also has important roles in regulating the acetylation of p53 [36, 48–54]. For example, HDAC1 and SIRT1 can abolish the acetylation of MDM2-mediated ubiquitination of p53 [48, 50, 52]. Western blot assay showed that KLF12 still reduced p53 protein levels in MCF-7 treated with HDACs and SIRTs inhibitors (Fig. S2h), which means KLF12 does not affect the ubiquitination of p53 by interfering with its deacetylation.

In the xenotransplantation experiment, the tumorigenicity of breast cancer cells was found to decrease when KLF12 expression in the cells was knocked down (Fig. 6a–c). Besides, patients with high KLF12 expression were those with a shorter relapse-free survival time (RFS) and poorer prognosis (Fig. 6d). Thus, the findings of this study indicated an important role for KLF12 in promoting human breast cancer growth and provided new insight into the underlying molecular mechanisms, with the p53/p21axis being a vital part of this mechanism. According to one previous study [55], p53 was found to down-regulate KLF12 expression by up-regulating the expression of microRNA-34a and microRNA-205. Moreover, the mRNA level of *KLF12* also gradually decreased with the increase of doxorubicin (DOX) treatment time in p53-positive cells. Therefore, there may be negative feedback regulation between KLF12 and p53, which still needs further verification.

**Fig. 7:**
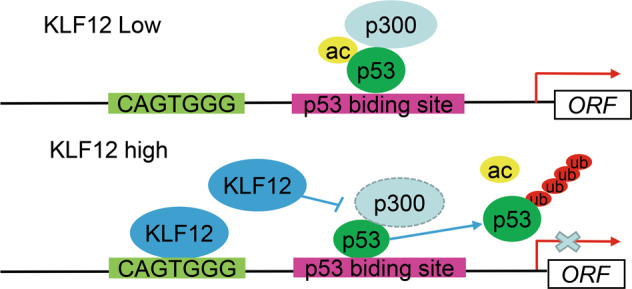
Schematic diagram of the molecular mechanism of KLF12 inhibiting *p21* transcription. When the KLF12 expression level is low, p53 interact with transcriptional coactivator p300 and acetylated by p300. Acetylation improves the transcriptional activation of p53 and stabilizes p53. When expression level of KLF12 is high, KLF12 inhibits the interaction between p53 and p300 and effectively prevents acetylation of p53, which promotes the ubiquitination of p53 and subsequent degradation. Ultimately, the ability of p53 to activate transcription of *p21* is reduced. Besides, KLF12 can bind to CAGTGGG motif and inhibit transcription of *p21*.

In conclusion, our datas suggest that KLF12 could inhibit *p21* transcription in breast cancer through two pathways. The first pathway is binding to the conserved binding site of the *p21* promoter and acting as a transcriptional repressor (Fig. 7); the second pathway is by inhibiting the transcriptional activity of p53 from interfering with the interaction between p53 and its co-activator p300 and effectively preventing the acetylation of p53 from promoting its ubiquitination and subsequent degradation (Fig. 7). The present research provides a new perspective on the treatment of breast cancer.

## Materials and methods

### Cell culture and transfection

HEK293T, ZR-75-30, MCF-7, and HeLa cells were purchased from China’s infrastructure of cell line resources; these cells were previously used in our other studies [56, 57]. HEK293T, MCF-7, and HeLa cells were maintained in DMEM supplemented with 10% FBS; ZR-75-30 cells were maintained in RPIM1640 supplemented with 10% FBS_._ All cells were in a humidified atmosphere containing 5%CO_2_/95% air at 37 °C. Cell transfection was carried out in a standard way using Lipofectamine 2000 (Invitrogen, Auckland, New Zealand).

### Plasmids and Antibodies

Human KLF12 was cloned from a human cDNA library using the forward primer 5′-CGGAATTCGAATATCCATATGAAGAG-3′ (underlined bases indicate EcoR I site) and reverse primer 5′-CGCGGATCCCACCAACATATGCCTC-3′ (underlined bases indicate BamH I site). The obtained KLF12 fragment was inserted into the expression vector pEGFP-C1 to yield pEGFP-C1-KLF12. Flag-tagged full-length KLF12 (Flag-KLF12) and Flag-tagged truncated KLF12 (KLF12Δ1 (1–144aa), Flag-KLF12 Δ2 (1–317aa) and Flag-KLF12Δ3 (145–402aa) were generated by standard PCR using specific primers with pEGFP-C1-KLF12 as the template. The PCR fragments were inserted into pCMV-N-Flag at the EcoRI-XhoI sites. The amplified full-length fragments of KLF12 were also inserted into pcDNA3.1-3HA and pGEX-4T-3 at the EcoRI-XhoI sites. GFP-p53, Flag-p53, and GFP-tagged truncated versions of p53 (GFP-p53Δ1 (1–97aa), GFP-p53Δ2 (98–292aa), GFP-p53Δ3 (293–393aa)), HA-48-Ub [58], HA-63-Ub [58], HA-p300 [59] and pGL3-p21 reporter construct (p21-Luc) were taken from the plasmid stock kept in our laboratory. p21-Luc contains approximately 1716 bp of sequence upstream of *p21* TSS. A series of truncated p21 promoters (−1670 to 1, −1582 to 1, −1376 to 1, and −1293 to 1) was also constructed according to a standard PCR-based cloning procedure using specific primers with p21-Luc as the template. The amplified promoter fragments were inserted into the pGL3-Luc luciferase reporter vector at the MluI-XhoI sites. KLF12-mut-p21-Luc was constructed by changing the KLF12 binding sequence 5′-CATGGGG-3′ into 5′-CACAAAG-3′ [60]. p53-mut-p21-Luc was constructed by changing the p53 binding sequence 5′-GAAGACTGGGCATGTCTGGGCAGAG-3’ into 5′-GAAGATGTTTACCGTCTGGGCAGAG-3′. KLF12-mut-p21-Luc and p53-mut-p21-Luc were constructed by following the instructions of the Quick-Change site-directed mutagenesis system (Stratagene) using p21-Luc as the template. The specific shRNA for KLF12 was inserted into the cloning vector pRNAT-U6.1/Hygro at the BamH I-HindIII sites. The KLF12 mRNA targeting sequence are as follows shKLF12#1: 5′-GCAATCGAATGAATAATCA-3′ [61], shKLF12#2: 5′-GACCTTAGATAGCGTTAA T-3′ [6]. The specific shRNA for p53 was from our laboratory. The effect of shp53 was verified by western blot. The p21 mRNA targeting sequences are as follows siRNAp21#1: 5′-AGACCAUGUGGACCUGUCATT-3′, siRNAp21#2: 5′-GACAGAU UUCUACCACUCCAATT-3′.

The primary antibodies were the following: anti-Flag (1:2000 for WB, F1804, Sigma-Aldrich, Saint Louis, Mo, USA), anti-Flag (1:2000 for WB, 1:500 for IP, F7425, Sigma-Aldrich, Saint Louis, Mo, USA), anti-MYC (1:2000 for WB, PLA0001, Sigma-Aldrich, Saint Louis, Mo, USA); anti-GFP (1:2000 for WB, TA150052, Origene, Rockville, MD, USA), anti-Myc (1:2000 for WB, TA150121, Origene, Rockville, MD, USA), anti-GFP (1:3000 for WB, 1:500 for IP, GTX113617, GeneTex, San Antonio, TX, USA), anti-HA (1:3000 for WB, 1:500 for IP, GTX115044, GeneTex, San Antonio, TX, USA), anti-GST (1:2000 for WB, ABN116, Millipore, Boston, MA, USA), anti-β-actin (C-2) (Santa Cruz, sc-9104, Dallas, CA, USA). Anti-GAPDH (GeneTex, GTX627408, Texas, USA), anti-GST (Millipore, ABN116, Boston, MA, USA), and anti-acetylated-lysine was purchased from Cell Signaling Technology (Cell Signaling Technology, #9441, Boston, MA, USA). anti-KLF12 (Santa Cruz,sc-134373, Dallas, CA, USA), anti-KLF12 (Abcam, Cambridge,ab129459, MA, USA), anti-p53 (Santa Cruz,sc-126, Dallas, CA, USA), and anti-p21 (Affinity Biosciences, #AF6290, Jiangsu, China).

### Luciferase reporter assay

Transcriptional activity was examined by a luciferase assay. Cells were seeded into a 24-well plate at a density of 1 × 10^5^ cells per well and cultured for 24 h. The cells were then transfected with the appropriate plasmid using Lipofectamine 2000 according to the manufacturer’s instructions. Three samples were used for each experimental group. Twenty-four hours after transfection, the cells were subjected to luciferase and Renilla activity assays using a Dual-Luciferase Reporter Assay system (Promega, Madison, WI, USA) according to the manufacturer’s instructions. Luciferase activity was measured and normalized against the β-galactosidase activity.

### RNA preparation and real-time qPCR

Total RNA was extracted from transfected MCF-7 cells using RNAiso reagents (Takara, Dalian, China). Quantitative PCR analysis was performed with the 2X SYBR Green qPCR master mix (Takara, Dalian, China) under standard conditions. Three samples for each experimental group. The primers used in the qPCR samples are shown in Table S1.

### Western blot, CoIP, ChIP, and ChIP-Re-ChIP assays

Western blot, co-immunoprecipitation (CoIP), chromatin immunoprecipitation (ChIP), and CHIP-Re-CHIP assays were conducted as previously described [62]. The primers for amplifying the p21 promoter used in ChIP and ChIP-Re-ChIP assays are shown in Table S2.

### Cell proliferation assay

MCF-7 cells or ZR-75-30 cells were cultured in a 96-well plate (2500 cells/well) for 24 h. MCF-7 cells were transfected with pEGFP-C1 or GFP-KLF12 for 1–5 days. ZR-75-30 cells were separately transfected with NC, shKLF12#1, or shKLF12#2 for 1–6 days. The medium was replaced with fresh medium daily for the cells transfected for more than 1 day. At the end of the transfection, 10 µl of MTT reagent (5 mg/ml) was added to each well containing the cells, followed by 4 h of incubation. After removal of the medium, 100 μl of DMSO was added to each well and properly mixed for another 10 min. The absorbance at 490 nm was determined using a microplate reader (Bio-Rad, CA, USA). Five samples were used for each experimental group.

A colony formation assay was carried out by plating 2000 cells in a six-well plate. Then, cells were cultured for 2 weeks and subjected to crystal violet staining as previously described. After staining, wells were washed three times with PBS and distained with acetic acid, and the absorbance of the crystal violet solution was measured at 590 nm using a microplate reader (Bio-Rad, CA, USA).

### Flow cytometric cell cycle and apoptosis assays

MCF-7 cells and ZR-75-30 cells were transfected with the appropriate plasmids for the cell cycle assay. After 48 hours of transfection, 1 × 10^6^ cells were harvested by centrifugation, washed three times with PBS, and fixed in 70% ethanol at 4 °C overnight. After that, the cells were again washed with cold PBS three times, re-suspended in 500 µl of propidium iodide (PI)/RNase staining solution (Sungene Biotech Co, Ltd., Tianjin, China), incubated for 30 min at 37 °C and then subjected to analysis by FACSCalibur (BD Biosciences, NJ, USA). The obtained data were analyzed with ModFit software (BD Biosciences, NJ, USA). Apoptosis of MCF-7 cells and ZR-75-30 cells treated with 20μm CDDP (Selleck, S1166, Texas, USA) for 16 h was assessed using the AnnexinV-FITC/PI Apoptosis Detection kit (Beyotime Biotechnology, C1062S, Shanghai, China) according to the manufacturer’s instructions. The data were collected by BD Accuri^TM^ C6 Plus Flow Cytometer (BD Biosciences, NJ, USA) and subsequently analyzed by the Flow Jo 10 (BD Biosciences, NJ, USA).

### Xenograft model

Five-week-old female athymic nude mice (BALB/C nude mice) were purchased from Changsheng Biotechnology Company (Dalian, Liaoning province, China). All the animals were housed in an environment with a temperature of 22 ± 1 °C, relative humidity of 50 ± 1%, and a light/dark cycle of 12/12 h. All animal studies (including the mice euthanasia procedure) were done in compliance with the regulations and guidelines of Dalian University of Technology institutional animal care and conducted according to the AAALAC and the IACUC guidelines.

All the mice were weighed and randomly divided into two groups according to body weight [random numbers were generated using the standard = RAND () function in Microsoft Excel] (5 mice per group): NC group and shKLF12#1 group. NC group was inoculated with ZR-75-30 cells transfected with NC; the shKLF12#1 group was inoculated with ZR-75-30 cells transfected with SHKLF12#1. Stably transfected ZR-75-30 cells were prepared in advance. First, the cells were digested with trypsin, then diluted to 1 × 10^5^ per 50 μl, and mixed well with the matrix adhesive (356234, Corning, USA) in a ratio of 1:1 and kept on ice. After the mice were anesthetized, each mice will be inoculated at right mammary fat pad with the mixture of cells and matrix adhesive. After 45 days, the nude mice were sacrificed, and the tumors were removed, measured, and photographed. The volume of each tumor was calculated using the standard formula *V* = 0.5 × *L* × *W*^2^, where *L* is the longest diameter and *W* is the shortest diameter.

### GST pull-down assay and immunofluorescence assay

GST alone, GST-KLF12 and GST-p53 were expressed in *Escherichia coli* BL21 (Takara, Dalian, China) and purified using Pierce GST Spin Purification Kit (Thermo Scientific, MA, USA) as per the manufacturer’s instruction. The purified GST and GST-KLF12 were seperately immobilized on a different Pierce Spin Column and used to pull down endogenous p53 from MCF-7 cell lysate. The purified GST and GST-p53 were seperately immobilized on a different Pierce Spin Column and used to pull down exogenous p300 from HEK293T cell lysate. An immunofluorescence assay was conducted as described previously [63].

### Statistical analysis

All experiments were repeated at least three times. Data were presented as mean ± SDs, and Student’s *t* test (unpaired, two-tailed) was used to compare two groups of independent samples. *p* < 0.01 and *p* < 0.05 indicated statistical significance.

### Reporting summary

Further information on research design is available in the Nature Research Reporting Summary linked to this article.

## Supplementary information

Supplementary Figure 1.

Supplementary Figure 2.

Supplementary legends

Table.S1

Table.S2

original western blot

Reporting Summary

## Data Availability

All datasets generated and analyzed during this study (luciferase reporter assay data, immunoprecipitation, and immunofluorescence assay data, xenograft model data, GST pull-down assay data, chromatin Immunoprecipitation assay data, cell proliferation assay data, cell cycle assay data) will be made available on reasonable request from the corresponding author Dr. Huijian Wu, email address: wuhj@dlut.edu.cn. Original full-length western blots are part of Supplementary Files.
