# Supplementary figures and images for: KLF12 promotes the proliferation of breast cancer cells by reducing the transcription of p21 in a p53-dependent and p53-independent manner
Source: Cell Death Dis. 2023 May 8;14(5):313. doi: 10.1038/s41419-023-05824-x (PMC10167366; doi:10.1038/s41419-023-05824-x)

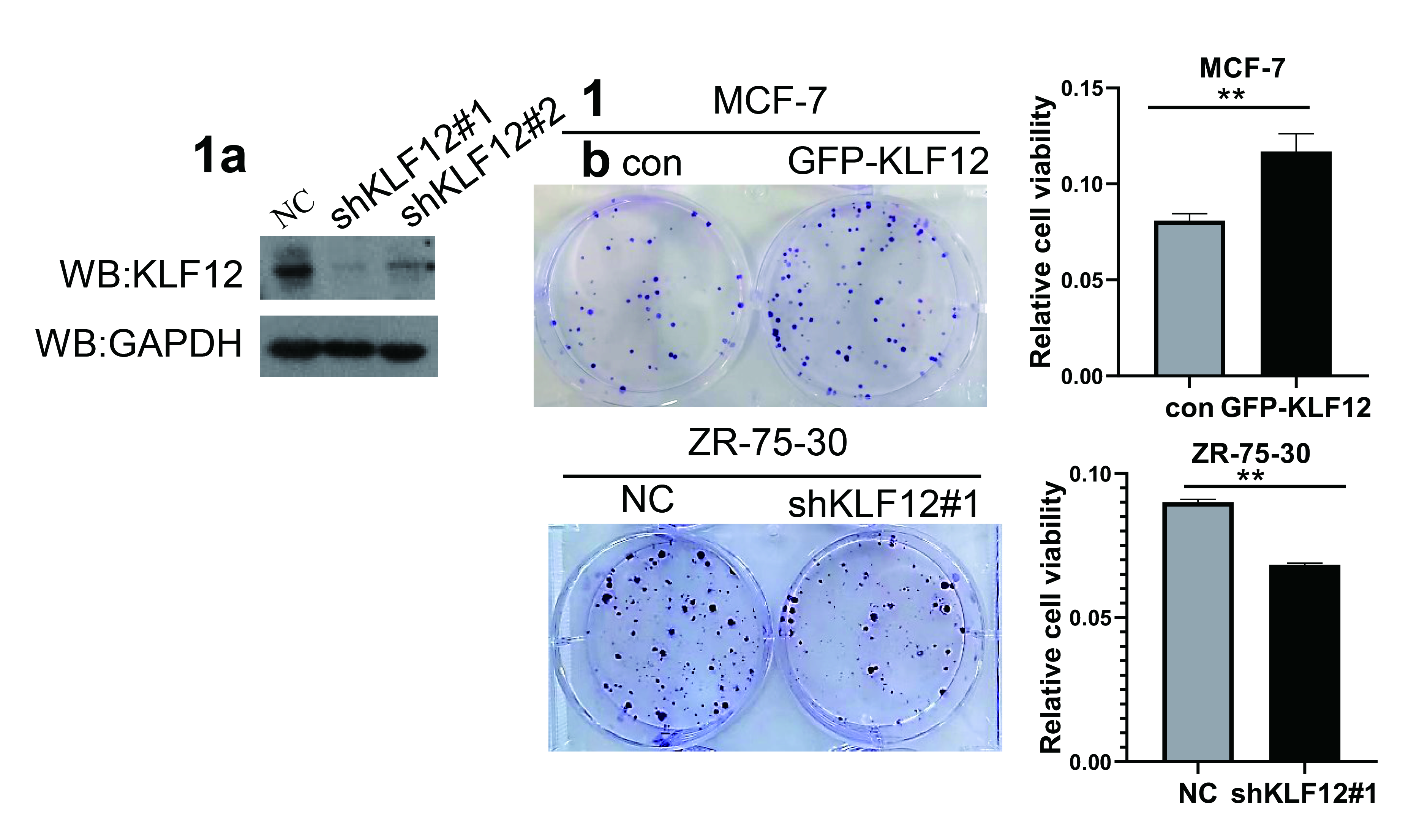

Supplement: Supplementary file 1 — Supplementary Figure 1. [file 41419_2023_5824_MOESM1_ESM.tif]

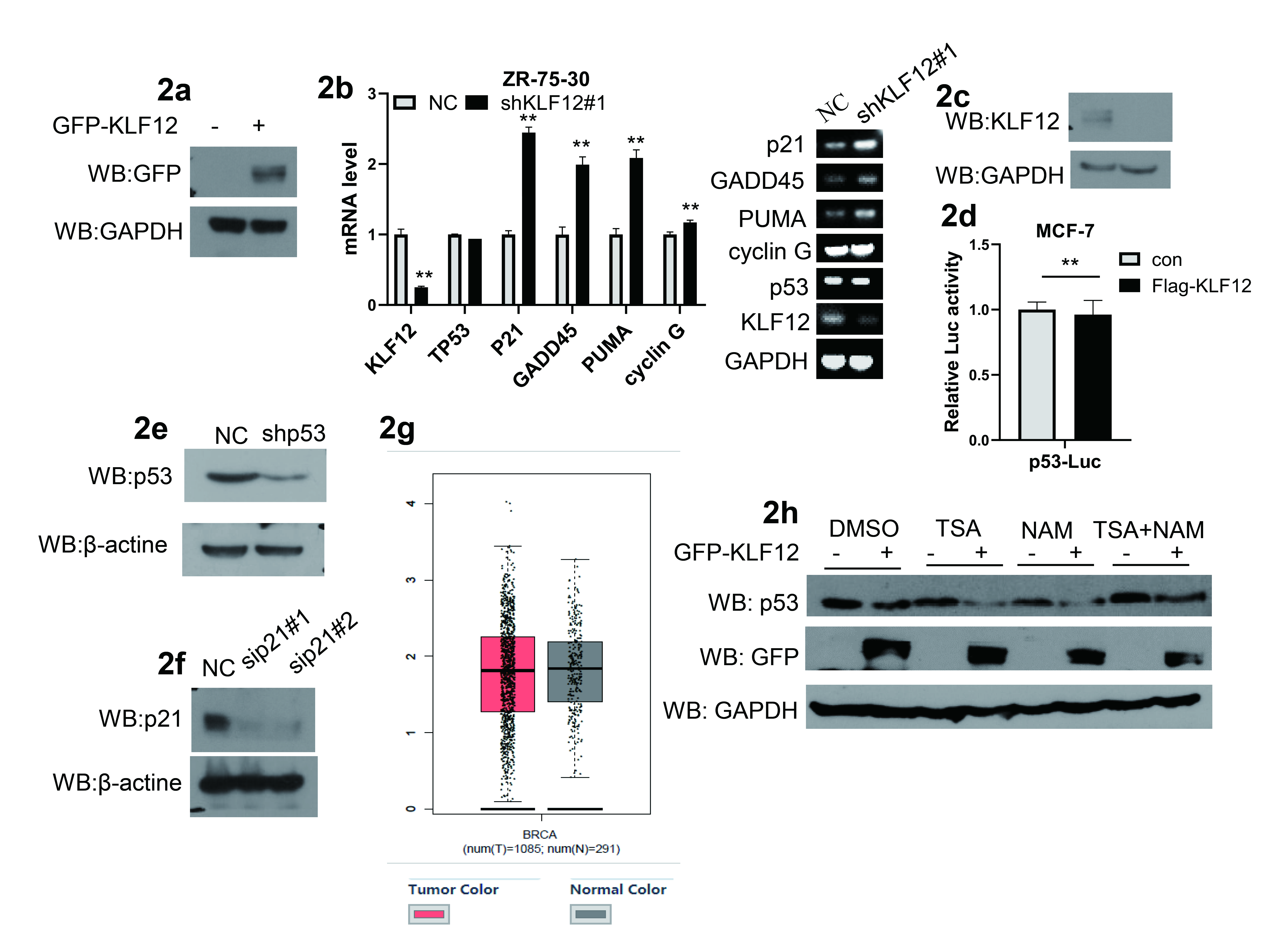

Supplement: Supplementary file 2 — Supplementary Figure 2. [file 41419_2023_5824_MOESM2_ESM.tif]
