# Supplementary material for: KLF12 promotes the proliferation of breast cancer cells by reducing the transcription of p21 in a p53-dependent and p53-independent manner
Source: Cell Death Dis. 2023 May 8;14(5):313. doi: 10.1038/s41419-023-05824-x (PMC10167366; doi:10.1038/s41419-023-05824-x)
Supplement: Supplementary file 3 — Supplementary legends [file 41419_2023_5824_MOESM3_ESM.docx]

**Supplementary Figure 1.** **(a)** Western blot assay detected KLF12 knockout efficiency in ZR-73-30 cells achieved by shKLF12 #1 and shKLF12 #2. **(b)** Colony formation assay detected the ability of colony formation of MCF-7cells and ZR-75-30 cells with the indicated plasmids transfected and the quantitative analysis of the ability of colony formation were shown (right).

**Supplementary Figure 2. (a)**Western blot assay detected the exogenous KLF12 in MCF-7 which was applied to the qPCR. **(b)** RT-PCR assay detected mRNA levels of p53 target genes CDKN1A, GADD45, PUMA, and cyclin G in ZR-75-30 cells transfected with NC, shKLF12#1. Data are presented as means ± SD. (p < 0.05, significant; ns, not significant; *, p < 0.05; **, p < 0.01). **(c)** Western blot assay detected KLF12 knockout efficiency in ZR-73-30 cells which was applied to the qPCR. **(d)**Luciferase reporter assay showed the effect of KLF12 on p53-Luc. For comparison, the p53-Luc activity level of the control cells was set to 1. Data are presented as means ± SDs (p < 0.05, significant; ns, not significant) **(e)** Western blot detected p53 knockout efficiency in MCF-7 cells achieved by shp53. **(f)** Western blot detected p21 knockout efficiency in ZR-75-30 cells achieved by sip21#1 and sip21#2. **(g)** Analysis of data from the GEPIA database (http://gepia.cancer-pku.cn) showing KLF12 expression in breast cancer versus normal breast tissues. **(h)** Western blot detected changes in p53 in MCF-7 cells in the absence or presence of KLF12 overexpression with the treatment of 1 mM Trichostatin A (TSA) or 5 mM nicotinamide (NAM). TSA and NAM are both acetylase inhibitors.
