# Supplementary material for: KLF12 promotes the proliferation of breast cancer cells by reducing the transcription of p21 in a p53-dependent and p53-independent manner
Source: Cell Death Dis. 2023 May 8;14(5):313. doi: 10.1038/s41419-023-05824-x (PMC10167366; doi:10.1038/s41419-023-05824-x)
Supplement: Supplementary file 6 — original western blot [file 41419_2023_5824_MOESM6_ESM.pdf]

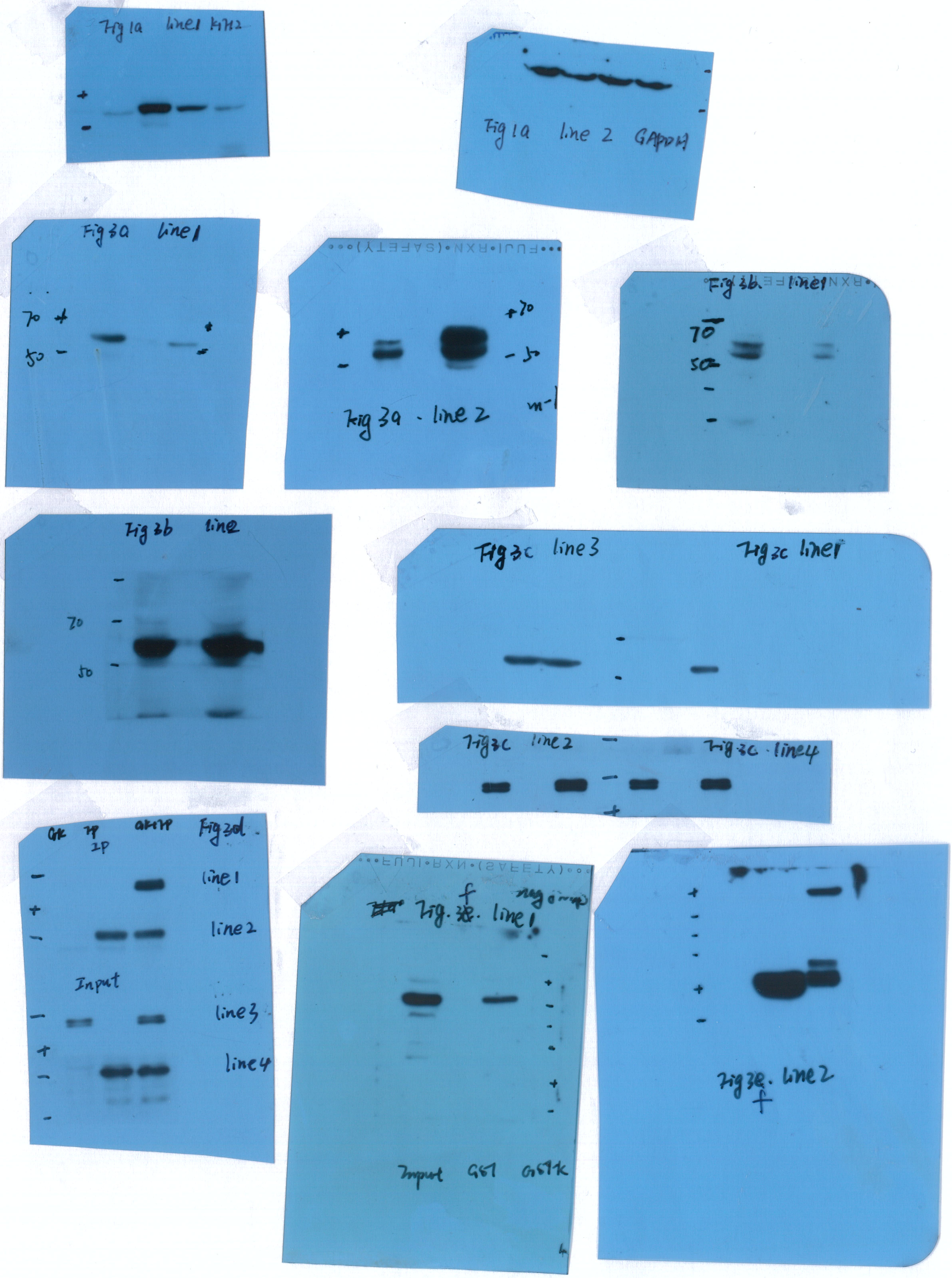
Fig 1a line1
Fig 1a line 2 GAPDH
Fig 3a line1
70
50
Fig 3a . line 2
70
50
Fig 3b line1
70
50
Fig 3b line2
70
50
Fig 3c line3
Fig 3c line1
Fig 3c line2
Fig 3c . line4
Fig 3d
line1
line2
Input
line3
line4
Fig. 3e. line1
Input
Fig 3e. line2

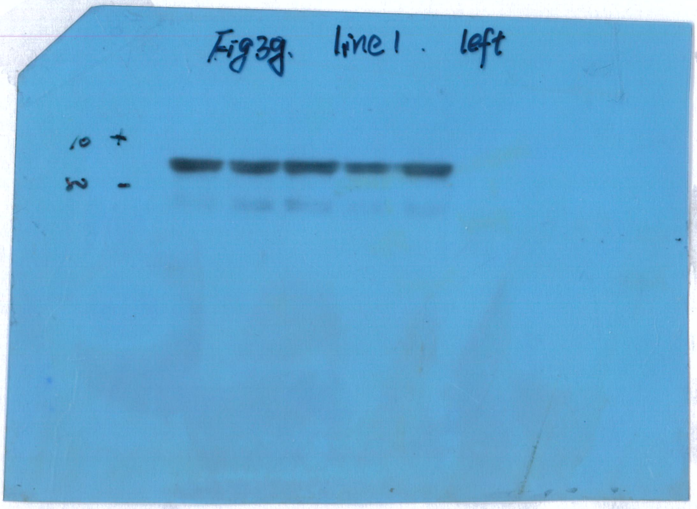
Fig3g. line1 . left
70
50

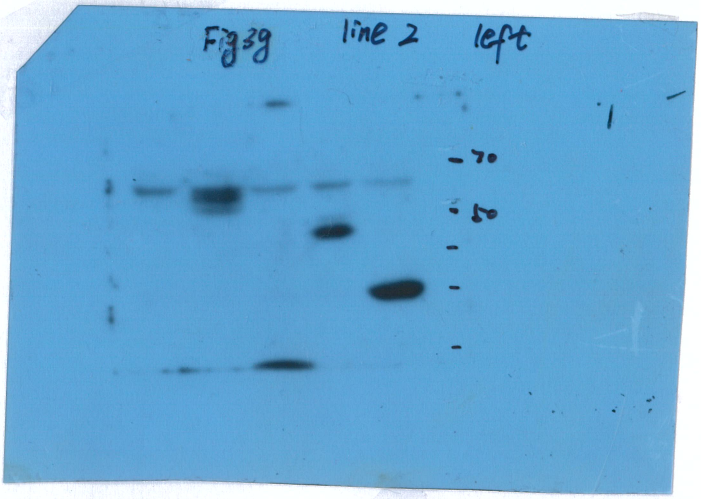
Fig3g line 2 left
70
50

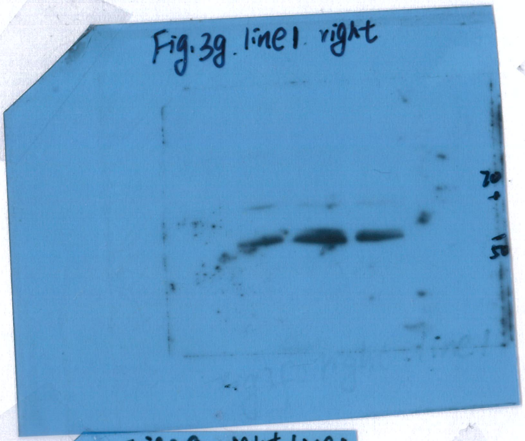
Fig.3g. line1 right
70
50

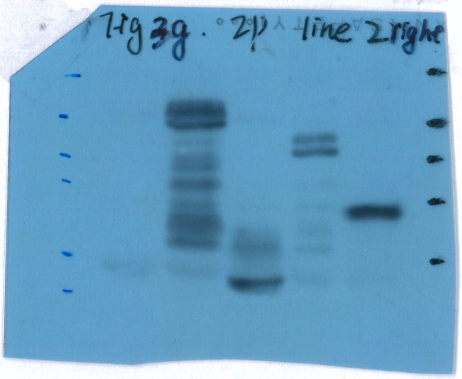
Fig 3g. line 2 right

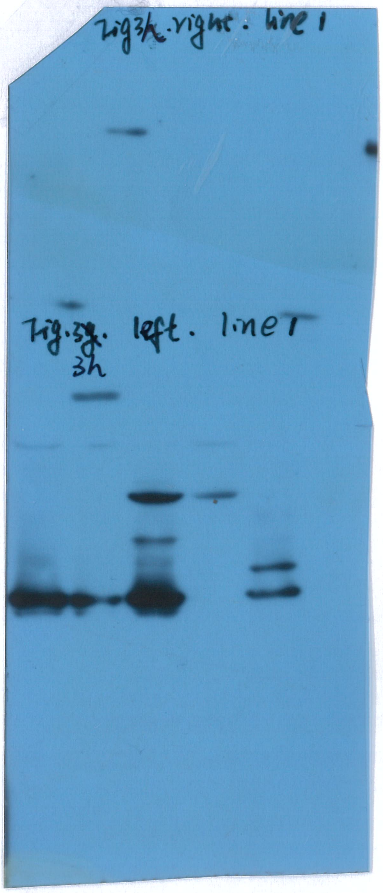
Fig3h. right . line 1
Fig.3g. 3h left . line 1

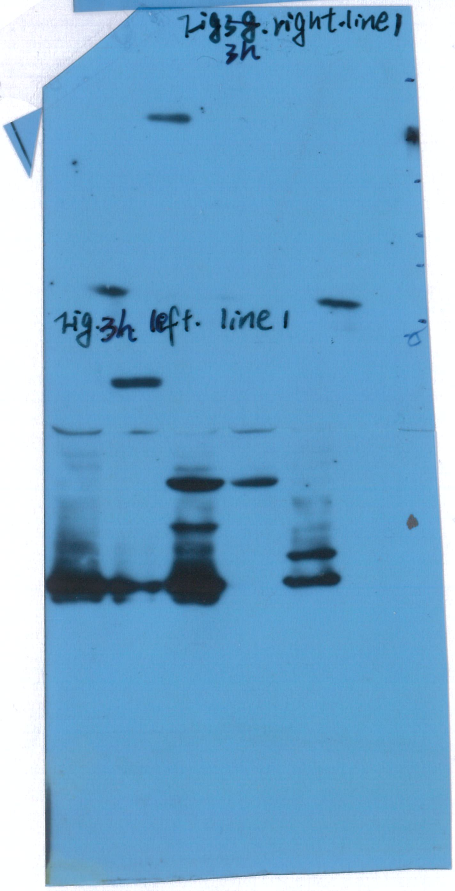
Fig3g. 3h right. line 1
Fig 3h left . line 1

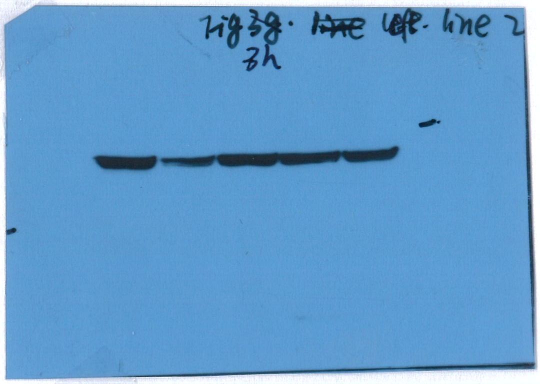
Fig3g. 3h left. line 2

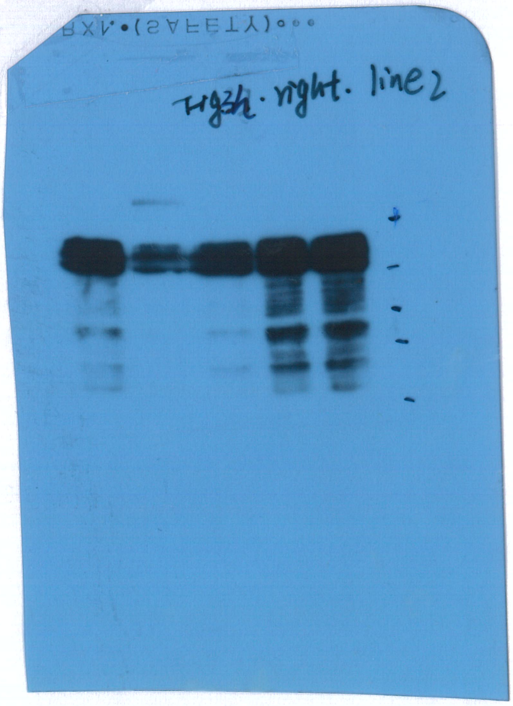
Fig3h. right. line 2

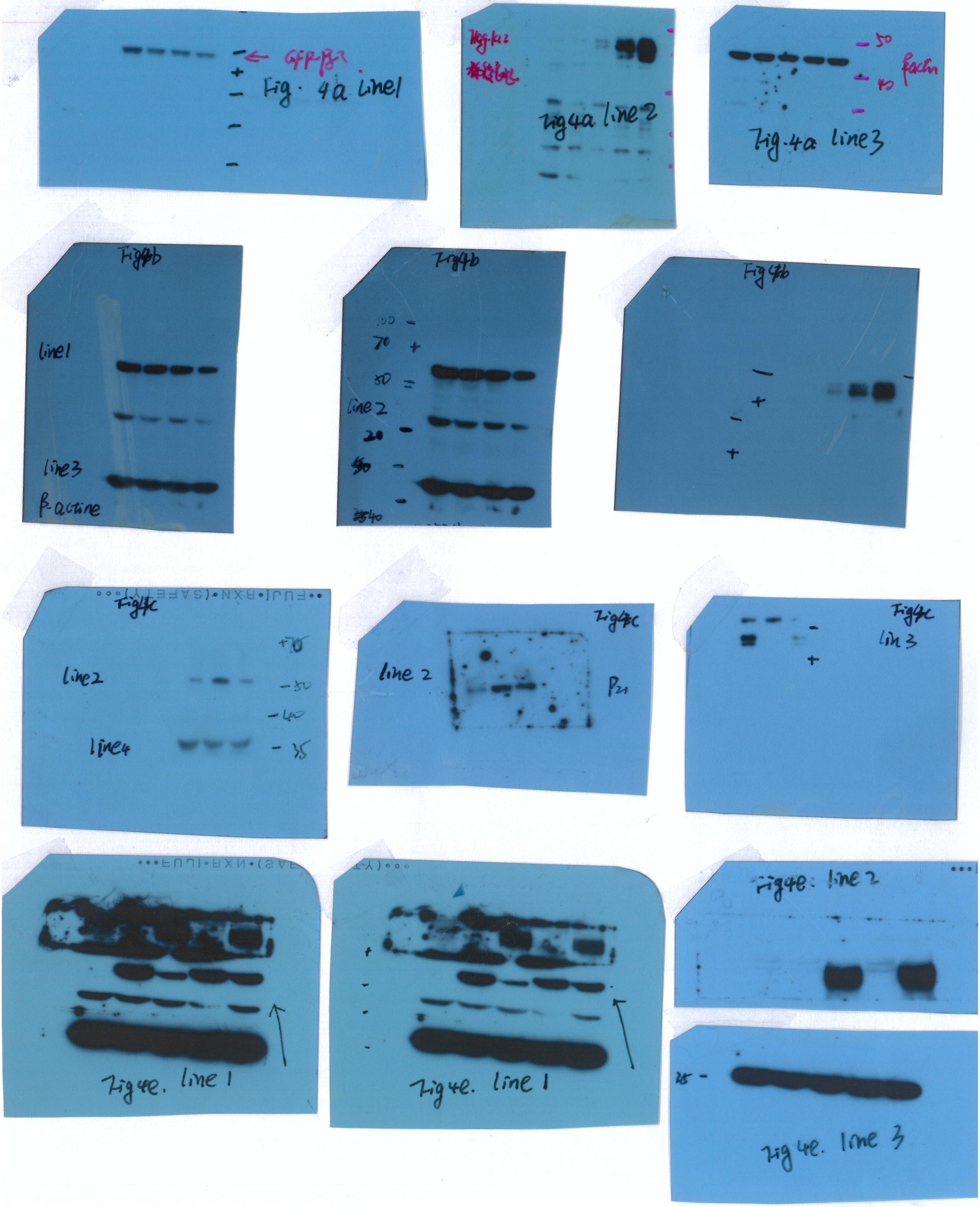
Fig. 4a Line1
Fig4a line2
Fig.4a line3
Fig4b
line1
line3
β-actine
Fig4b
line2
Fig4b
Fig4c
line2
line4
Fig4c
line 2
P21
Fig4c
lin3
Fig4e. line1
Fig4e. line1
Fig4e: line2
Fig4e. line 3

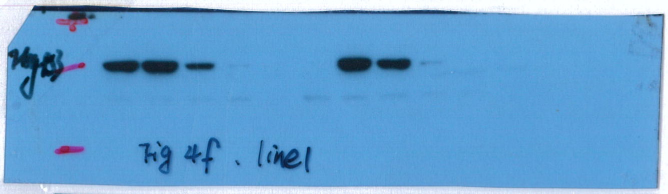
Fig 4f . line1

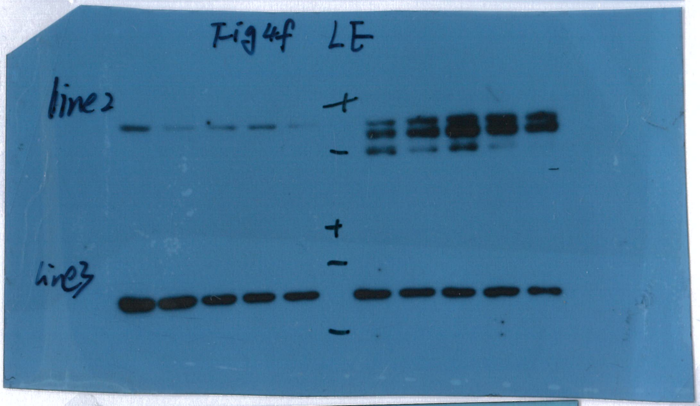
Fig 4f LE
line2
line3

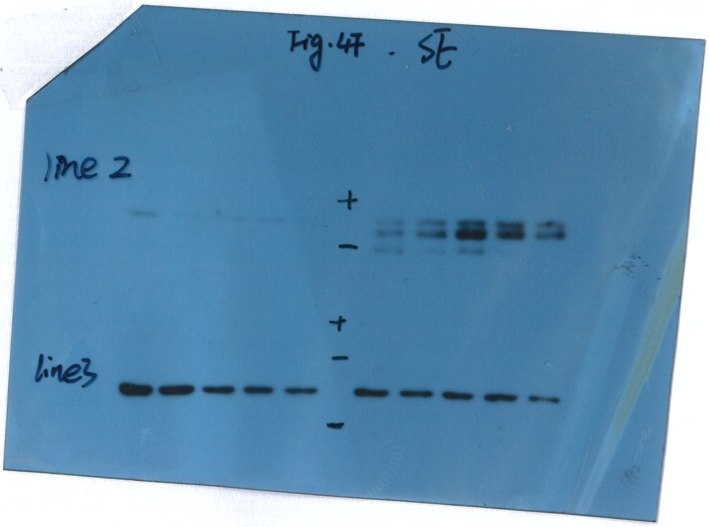
Fig.4f . SE
line 2
line3

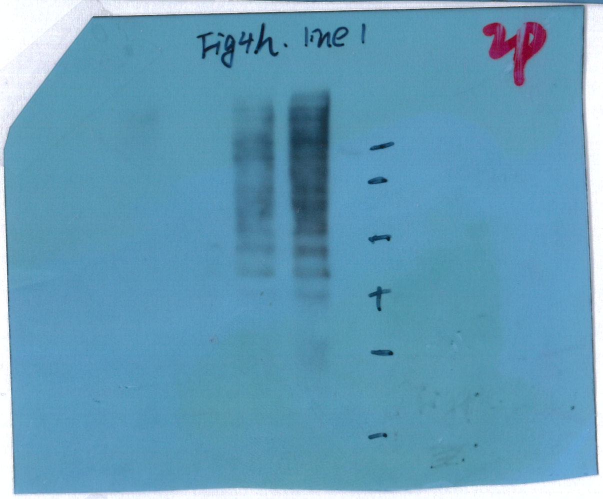
Fig4h. line 1

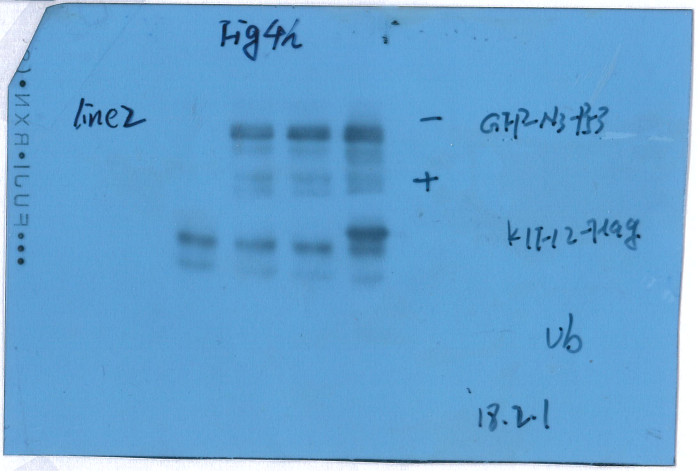
Fig4h
line2
Ub
18.2.1

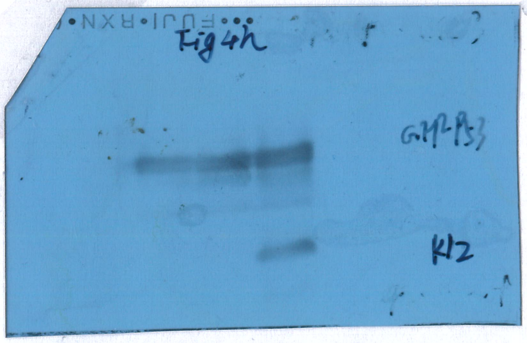
Fig4h

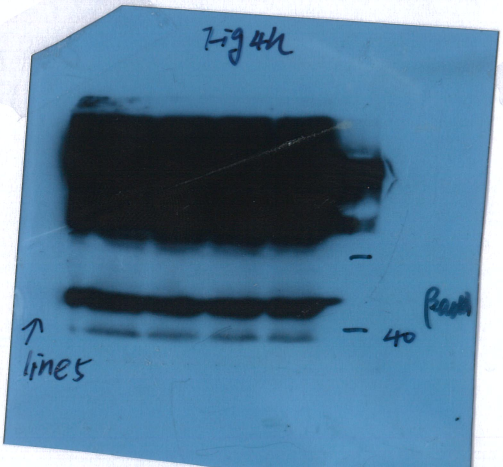
Fig4h
40
line5

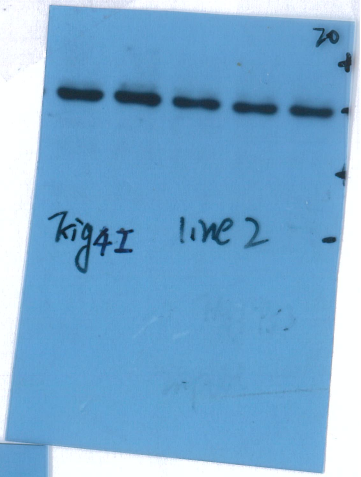
70
Fig4I line 2

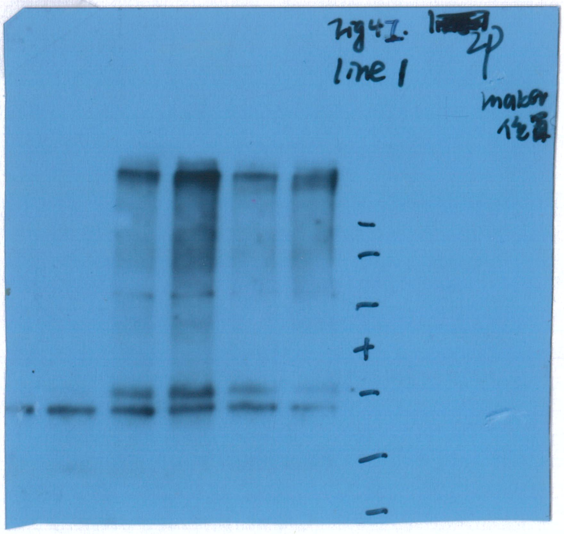
Fig4I.
line 1

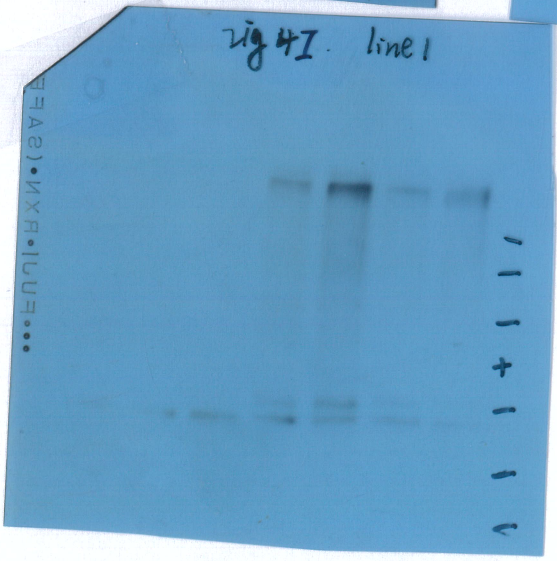
Fig 4I . line 1

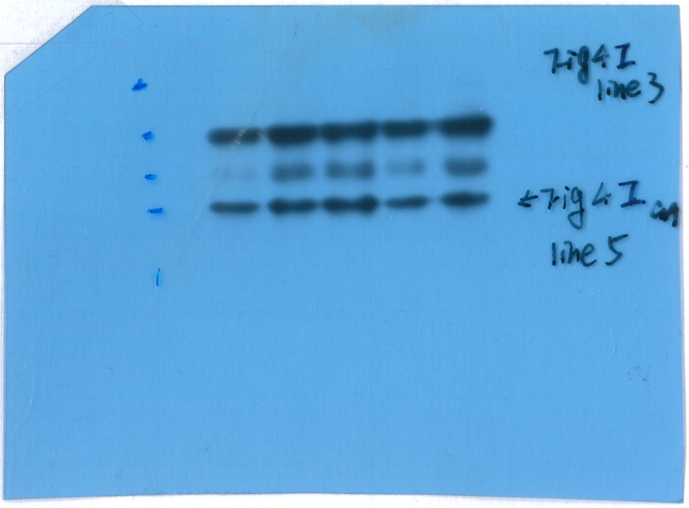
Zig4I
line 3
←Zig4I
line 5

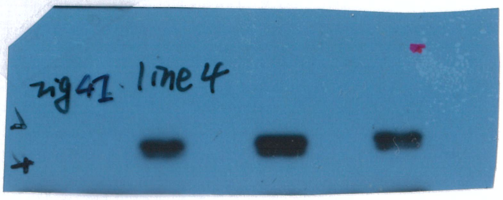
Zig4I line 4

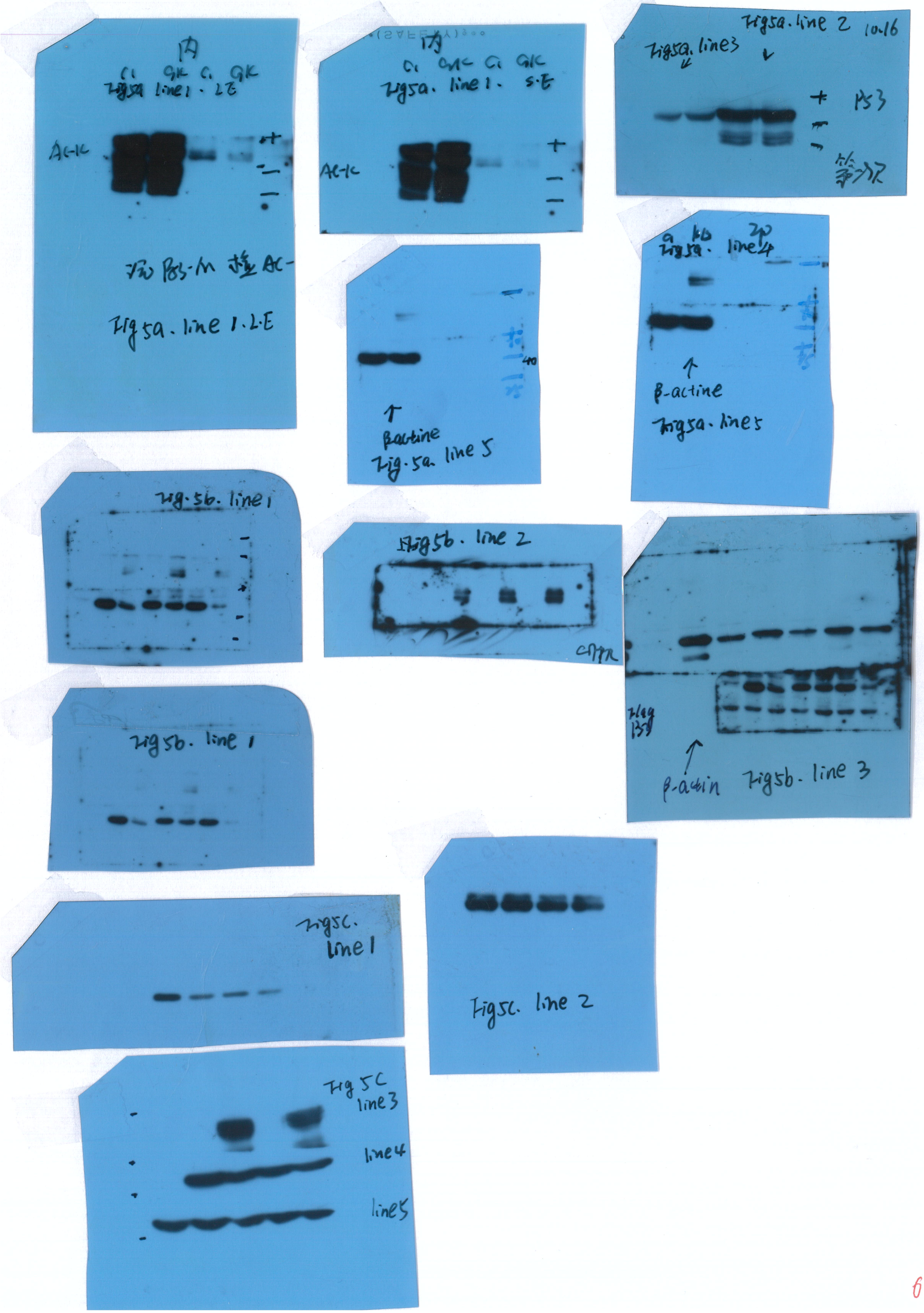
Ac-K
Fig5a. line 1.2.E
Ac-K
Fig5a. line 1. S.E
Fig5a. line 2
10.16
Fig5a. line3
P53
β-actine
Fig.5a. line 5
β-actine
Fig5a. line5
Fig.5b. line1
Fig5b. line 2
β-actin
Fig5b. line 3
Fig5b. line 1
Fig5c. line1
Fig5c. line 2
Fig 5C line3
line4
line5

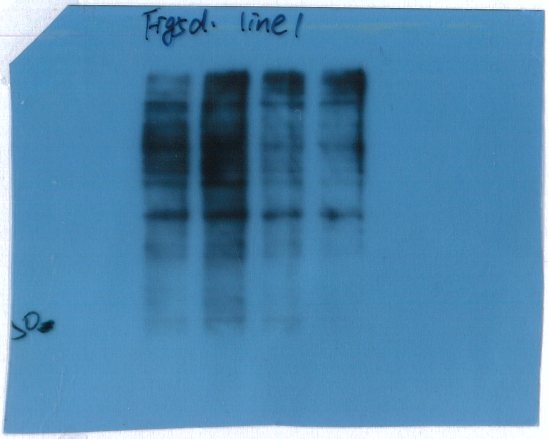
Fig5d. line1
50

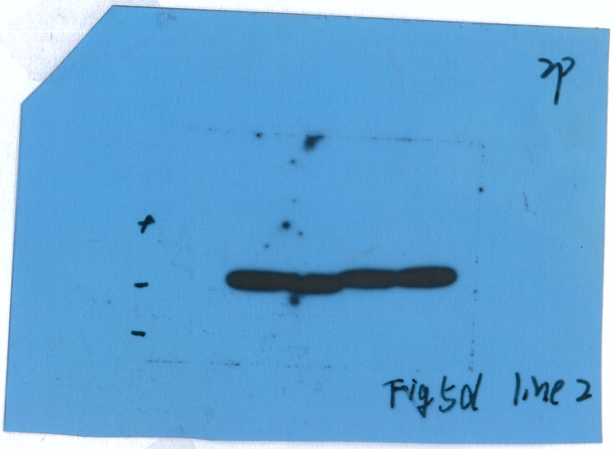
IP
Fig5d line 2

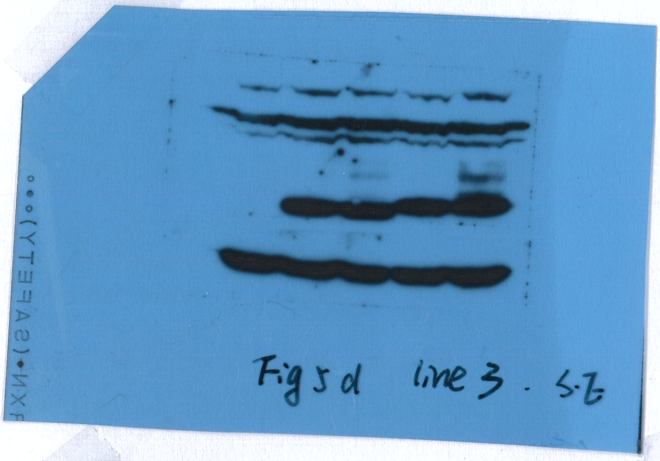
Fig 5d line 3 . S-Z

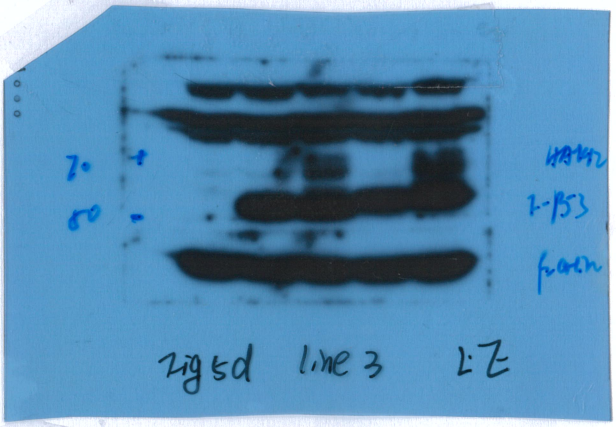
70
50
F-p53
Fig5d line 3 L-Z

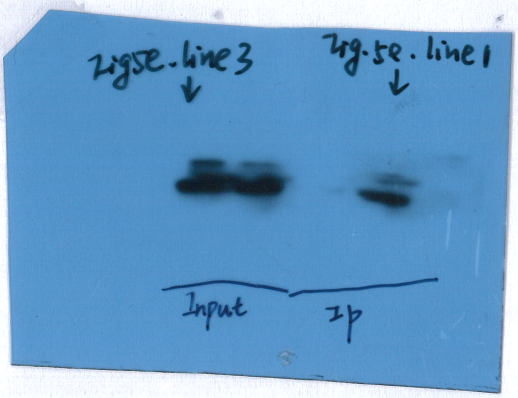
Fig5e. line3
Fig.5e. line1
Input
IP

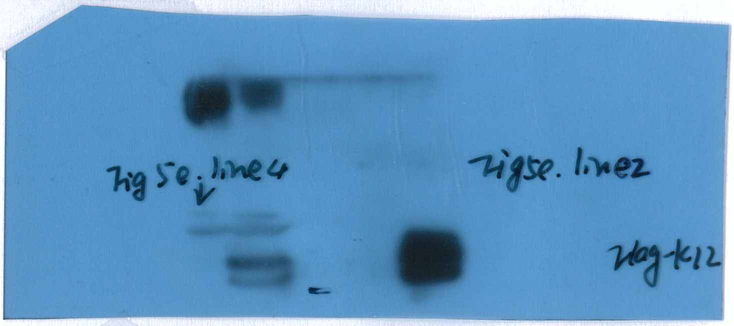
Fig5e. line4
Fig5e. line2
Flag-K12

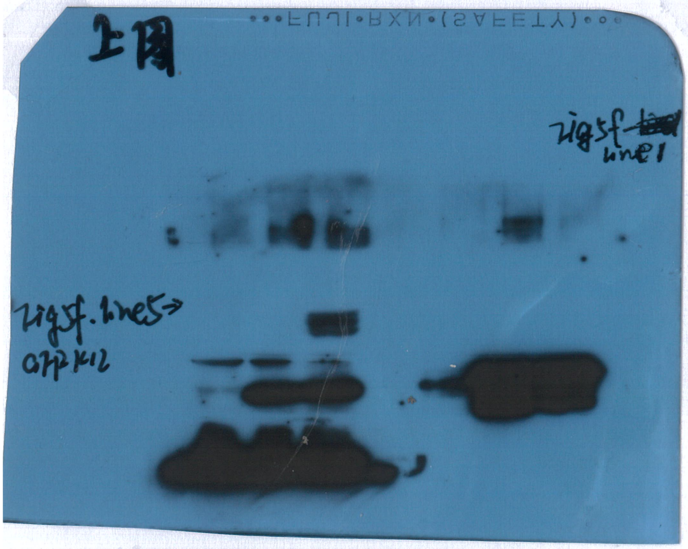
上图
Fig5f line1
Fig5f. line5

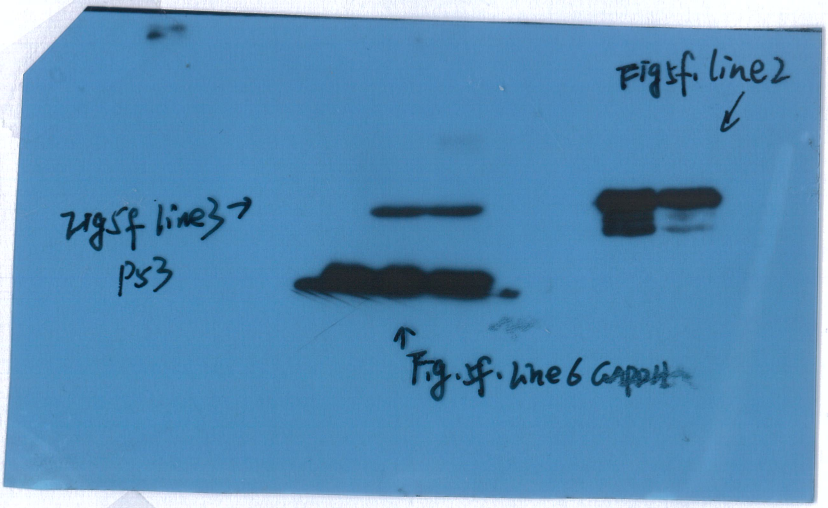
Fig5f. line2
Fig5f line3
p53
Fig.5f. Line6 GAPDH

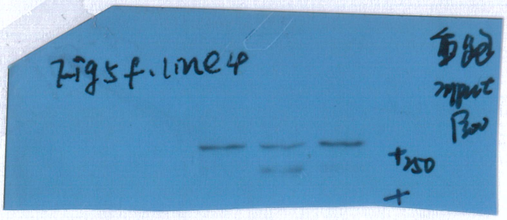
Fig5f. line4
Input

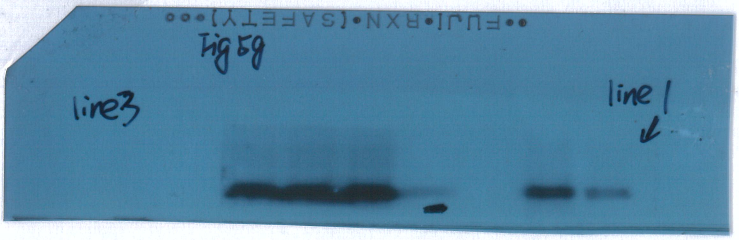
Fig5g
line3
line1

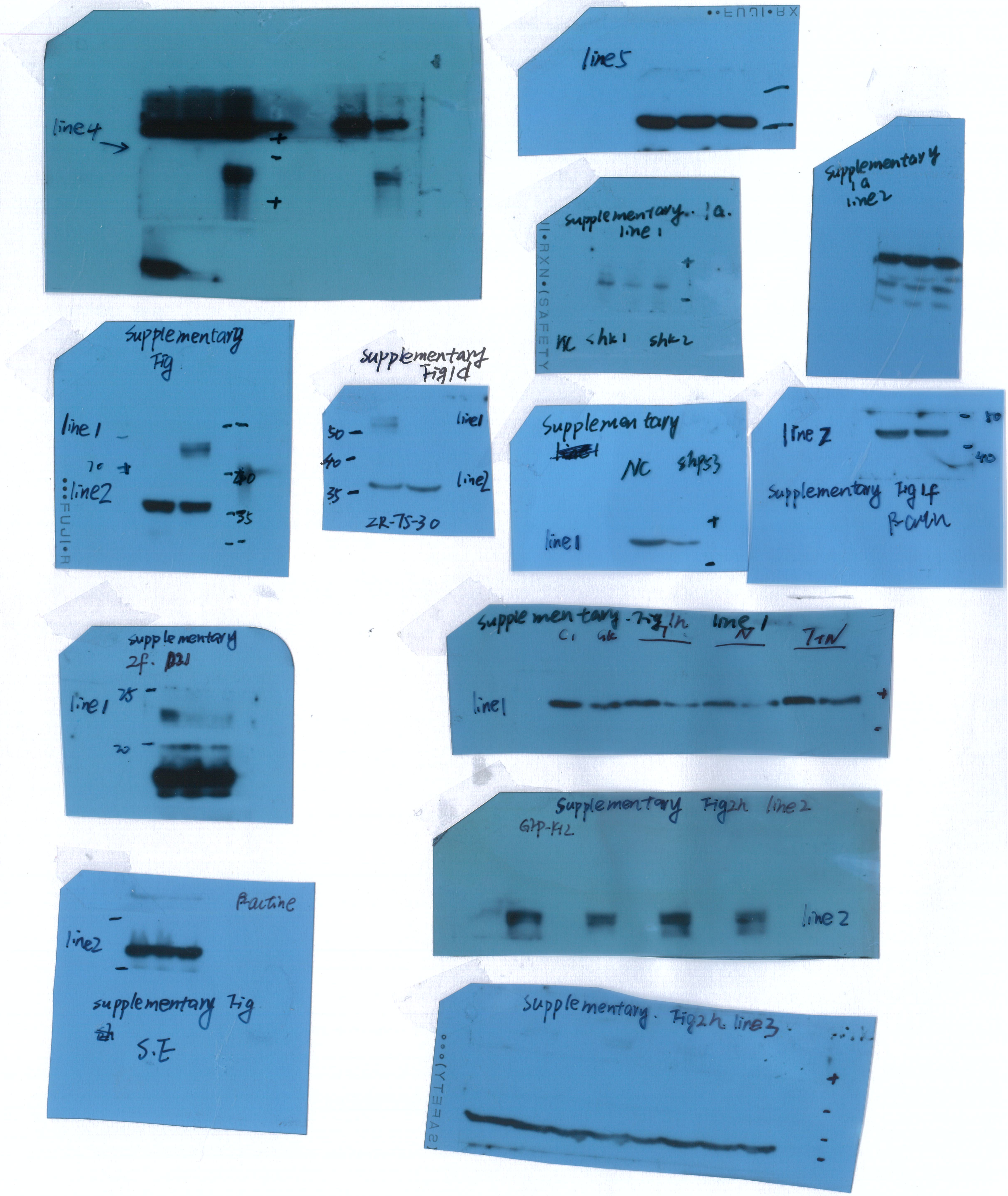
line4
+
-
+
line5
supplementary 1a.
line 1
NC shk1 shk2
+
-
supplementary 1a
line2
supplementary Fig
line1
70
line2
35
supplementary Fig1d
50
40
35
line1
line2
ZR-75-30
supplementary
NC shp53
line1
+
-
line 2
80
40
supplementary Fig1f
β-actin
supple mentary Fig1h line 1
line1
supplementary 2f.
line1
25
20
supplementary Fig2h line2
GFP-Ki2
line 2
β-actine
line2
supplementary Fig
S.E
supplementary. Fig2h. line3
+
